# Meningococcal vaccine 4CMenB elicits a robust cellular immune response that targets but is not consistently protective against *Neisseria gonorrhoeae* during murine vaginal infection

**DOI:** 10.1128/msphere.00940-24

**Published:** 2025-04-16

**Authors:** Joseph J. Zeppa, Jamie E. Fegan, Pauline Maiello, Epshita A. Islam, Isaac S. Lee, Christine Pham, Laura-Lee Caruso, Scott D. Gray-Owen

**Affiliations:** 1 Department of Molecular Genetics, Temerty Faculty of Medicine, University of Toronto 204248, Toronto, Ontario, Canada; 2 Department of Microbiology and Molecular Genetics, University of Pittsburgh School of Medicine 541988, Pittsburgh, Pennsylvania, USA; 3 Center for Vaccine Research, University of Pittsburgh School of Medicine 588296, Pittsburgh, Pennsylvania, USA; 4 Department of Biochemistry, University of Toronto 233836, Toronto, Ontario, Canada; University of Wyoming College of Agriculture Life Sciences and Natural Resources, Laramie, Wyoming, USA

**Keywords:** *Neisseria gonorrhoeae*, 4CMenB, Bexsero, T-cell immunity, adaptive immunity

## Abstract

Retrospective epidemiological studies suggest that the licensed serogroup B meningococcal vaccine 4CMenB (Bexsero) provides some protection against the closely related pathogen *Neisseria gonorrhoeae* in humans. This result has been replicated in murine models of gonococcal colonization, with a gonococci-reactive humoral response and more rapid clearance of vaginal infection. However, immunization with 4CMenB consistently elicits a robust humoral response but does not protect all individuals; hence, the correlates of protection remain undefined. Herein, we exploit the fact that 4CMenB promotes gonococcal clearance in only a subset of immunized mice to perform a broad analysis of the adaptive response in animals that are or are not protected. We observe that 4CMenB vaccination induces high levels of anti-neisserial antibodies in both serum and the vaginal lumen, and a robust cellular response highlighted by an increase in both conventional naïve and memory populations as well as unconventional lymphocyte subsets. Multiplex and flow cytometry results show that 4CMenB vaccination generates a robust, multi-faceted cytokine response that spans numerous T cell subsets (T_H_1, T_H_2, T_reg_, and T_H_17) and that non-T non-B lymphocytes play an important role in this response, as indicated by an unbiased principal component analysis. Together, this work provides the first comprehensive analysis of the robust humoral and complex cellular response to 4CMenB so as to reveal the effector mechanisms that may contribute to immunity against vaginal gonococcal infection.

**IMPORTANCE:**

Gonorrhea, a sexually transmitted infection caused by the human-specific pathogen *Neisseria gonorrhoeae* (Ngo), is a growing public health concern due to its rise in prevalence and increasing antibiotic resistance against first-line agents. There is currently no vaccine available for this important bacterium due, in part, to our lack of understanding of immune correlates of protection. Interestingly, a human-approved vaccine (4CMenB; Bexsero) against a related pathogen (*N. meningitidis*; a cause of meningitis) has demonstrated some protection against gonorrhea in epidemiologic studies. Herein, we provide the first detailed analysis of cellular and antibody-mediated immune responses to this vaccine in animals protected against gonococcal colonization. These findings provide new understanding regarding immune correlates of protection against *N. gonorrhoeae*, providing new insight into immune protection and helping guide the development of a much-needed vaccine.

## INTRODUCTION

*Neisseria gonorrhoeae* (Ngo), which causes the sexually transmitted infection gonorrhea, is a human-restricted bacteria responsible for over 82 million new infections globally in 2020 (1). Although still treatable with antibiotics, the emergence and rapid evolution of antimicrobial-resistant strains is raising the possibility of untreatable gonorrhea (2). Uncomplicated urogenital infection by Ngo does not typically lead to the generation of a protective immune response, allowing for repeated infections (3). Due to the absence of immunity, it remains unknown as to what immune responses can confer protection, and thus, it remains unclear what type of correlates of protection can be used to guide the development of a new gonococcal vaccine (4).

The four-component meningococcal vaccine 4CMenB (marketed as Bexsero by GSK) is a licensed vaccine that was developed to protect against serogroup B strains of the pathogen *Neisseria meningitidis* (Nme) (5). This multicomponent vaccine is comprised of outer membrane vesicles (OMVs) from Nme strain NZ98/254 mixed with recombinant proteins’ neisserial heparin-binding antigen (NHBA), neisserial adhesin A (NadA), and factor H binding protein (fHbp), along with two proteins fused to NHBA and fHbp (GNA1030 and GNA2091, respectively) to increase bactericidal titers (6). Serological analysis of individuals who have been immunized with this vaccine has identified antibodies that cross-react with Ngo antigens that are conserved between these organisms (7, 8), including OMV-derived antigens and the recombinant NHBA, GNA2091, and GNA1030, although the latter two are not believed to be surface exposed in Ngo (7). Even more notable is that several epidemiological studies have found that individuals vaccinated with 4CMenB have a reduced incidence of Ngo infection (9–11). Vaccine effectiveness is estimated to range between 33% and 44% in humans, a finding that has recently prompted some governments to recommend vaccinating individuals at high risk of contracting gonorrhea with 4CMenB (12).

Given the finding that 4CMenB confers some protection against Ngo infection in humans, it is satisfying that this vaccine also confers protection against gonococcal infection (13) in a well-established mouse vaginal infection model (14). In this case, 4CMenB accelerated gonococcal clearance from the lower genital tract and elicited cross-reactive antibodies to some of the same gonococcal components recognized by 4CMenB-immune human serum (15), supporting the relevance of this model for further investigation of vaccine-induced mechanisms of protection. In this study, we have taken a comprehensive approach to analyze the cellular and humoral immune responses of each individual animal to identify potential immune correlates of protection. Enzyme-linked immunosorbent assays (ELISAs), western blots, and serum bactericidal assays were performed to compare the humoral response against Ngo in control versus vaccinated animals that were or were not protected; flow cytometry was performed on spleen and genital tract lymphocytes to assess immune populations and cellular cytokine responses; and multiplex assays were performed to assess cytokines and chemokines from vaccine-stimulated lymphocytes from the vaginal lumen prior to and during gonococcal infection, as well as genital tract tissues and spleen at endpoint. Finally, principal component analyses were performed on all data sets to determine what factors were driving the variability in our data and determine whether immune correlates of protection could be revealed by this study. This unbiased approach demonstrates that the vaccine elicits a consistently high humoral response that does not explain the different levels of protection in these animals and reveals effector mechanisms that may combine to contribute to immunity against vaginal infection by Ngo.

## RESULTS

### 4CMenB reduces the duration of colonization and bacterial load of *N. gonorrhoeae* in a murine vaginal infection model

To establish that the previously reported protection that 4CMenB conferred against mouse vaginal colonization by Ngo could be replicated in our study, we performed a preliminary vaccination and challenge experiment by vaginal inoculation of female BALB/c mice to monitor protection and allow us to power this study. In this pilot study, nine alum (control) and 10 4CMenB vaccinated mice were followed for 10 days post-infection to assess the ability of the treatment group to clear the gonococci sooner. Consistent with the previous work, we saw a significant reduction in the percentage of colonized mice that were 4CMenB vaccinated compared with the control group. Based upon this, we powered our study using percent colonization data from day 6 because it was the first day that we observed equivocal protection data between our Ngo infection model and that observed in human retrospective studies (approximate vaccine efficacy of 40%), and we wanted to identify early effectors of protection rather than monitor immunity post-challenge. Using a Type I error rate of 0.05 and 80% power, it was determined that we would need a minimum sample size of 14 animals per group to detect a statistical difference in colonization. Additionally, to reduce the chance of experimental error, our study was performed two times independently, and the results were pooled to reach our final group sizes. This balance of animals also gave us the opportunity to perform complex cellular analyses to search for potential immune correlates of protection as a secondary analysis.

**Fig 1:**
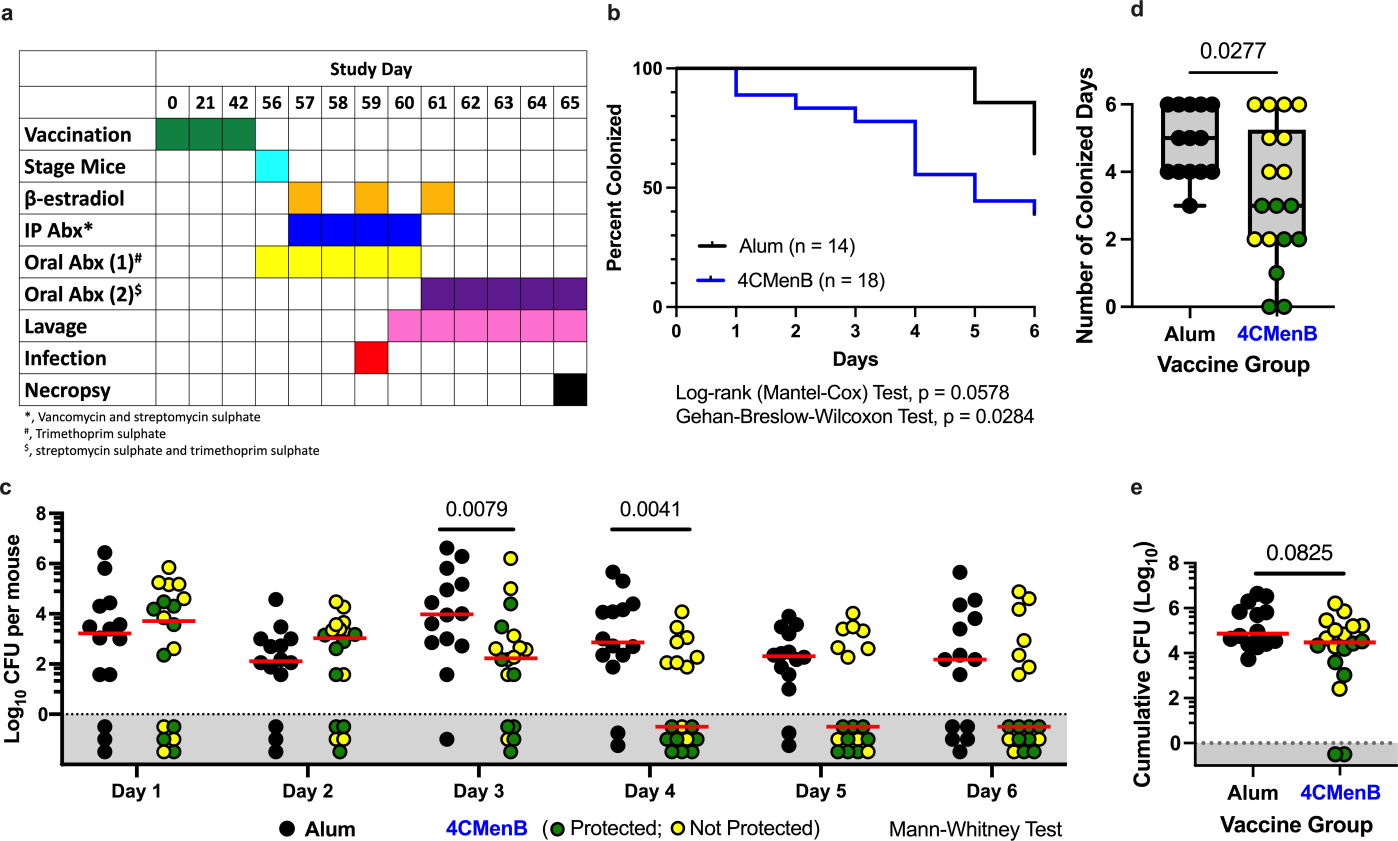
4CMenB vaccination protects mice from a vaginal gonococcal challenge, speeds up clearance, and reduces the bacterial load. (a) The vaccination and challenge study outline. (b) Kaplan-Meier curve showing the percentage of animals colonized in each group (4CMenB, blue line; alum control, black) on each day post-challenge. *P* values for log-rank (Mantel-Cox) and Gehan-Breslow-Wilcoxon statistical tests are shown. (c) The vaginal bacterial load recovered from each animal on each day. (d) The total number of days colonized for each animal. The box plot denotes the median and upper/lower quartiles within the gray area with the whiskers extending to the minimum and maximum values. (e) The cumulative bacterial load recovered from each animal throughout the experiment. Each symbol represents one animal. The alum control group is shown in black. The 4CMenB-vaccinated group is either blue (**b**) or separated into green circles (4CMenB-vaccinated protected; no bacteria were recovered from vaginal lavages on at minimum days 4–6) or yellow circles (not protected 4CMenB-vaccinated animals; bacteria recovered from vaginal lavage on one or more of the last 3 days of the experiment) (c–e). Red horizontal lines denote the median, and symbols within the gray area denote no Ngo recovery (**c and e**). Mann-Whitney statistical test was performed, and *P* values are listed (c–e).

The mice were randomized 1:1 into either alum control or 4CMenB group, vaccinated at days 0, 21, and 42 and then infected with approximately 1 × 10^7^ colony-forming units (CFU) of Ngo strain FA1090 after estradiol treatment. Vaginal lavages were collected daily to assess colonization and enumerate bacterial burden (Fig. 1a). As in our pilot study, a statistically significant reduction in the percentage of animals colonized was seen (*P* = 0.0284; Fig. 1b). Comparison of vaginal bacterial load between 4CMenB and alum on each day also revealed a significant reduction in bacterial burden in 4CMenB-vaccinated animals on days 3 and 4 post-infection (*P* = 0.0079 and 0.0041, respectively; Fig. 1c). We also observed a statistically significant reduction in the number of CFU positive days in our 4CMenB group (*P* = 0.0277; Fig. 1d); however, the cumulative CFU was not statistically different (*P* = 0.0825; Fig. 1e) but was trending lower in the 4CMenB group. The results of both independent studies are shown in Fig. S1. Within the 4CMenB group, it was noted that there were approximately 44% (8/18) of animals that had no bacterial recovery after day 3 post-infection; we labeled these as “protected” animals. When this group was separated from the “not protected” 4CMenB-immunized animals and compared with the alum control group, it was noted that the “protected” group had statistically fewer days colonized and lower cumulative CFU, whereas the unprotected animals were not statistically different from controls (Fig. S2a and b). We also noted a significant correlation (*P* = 0.0001; R^2^ = 0.3971) between both the number of colonized days and cumulative CFU between the protected and all other animals in the study (Fig. S2c).

### 4CMenB vaccination elicited significant anti-neisserial antibody titers with notable mouse-to-mouse variability in the bacterial components recognized

To compare the humoral response between our two groups, ELISAs were performed using pre-infection (post-vaccination) serum and vaginal lavage samples against whole cell heat-inactivated Nme NZ98/254 (the parental strain of the OMVs in 4CMenB) and Ngo FA1090 (the challenge strain used in this study). As expected, we observed a significantly higher serum IgG titer against both bacterial strains (*P* = 0.0001) in our 4CMenB-vaccinated animals compared with control (Fig. S3a and b), though titers against the Ngo challenge strain were approximately 20 times lower than they were against Nme (median values of 61.29 µg mL^−1^ and 1,238.93 µg mL^−1^, respectively). For vaginal IgA, titers against the meningococcal strain were significantly higher in 4CMenB-immunized animals (*P* = 0.0289), but titers against the challenge gonococcal strain were not (Fig. S3c and d). Additionally, there was no difference in titers observed between our protected and not protected 4CMenB animals (Fig. S3). We also noted that there was no correlation between total serum IgG or mucosal IgA antibody titers and the cumulative CFU or the number of colonized days (Fig. S4).

To further categorize the IgG response, IgG isotypes in terminal serum against inactivated Nme NZ98/254 and Ngo FA1090 were quantified. Significant binding of all four isotypes (IgG1, IgG2a, IgG2b, and IgG3) was detected for the 4CMenB group against Nme NZ98/254 (Fig. S5a through d, *P*<0.0001), and significantly, albeit lower, IgG1, IgG2a, and IgG2b levels were detected against Ngo FA1090 (Fig. S5e through h, *P*<0.0001). Notably, IgG2a and IgG2b were the predominant classes bound to Nme NZ98/254, whereas cross-reactive antibodies against Ngo FA1090 were mainly IgG1. A negative correlation between IgG2b signal against Nme NZ98/254 was observed for both the number of colonized days (*P* = 0.0332) and cumulative CFU (*P* = 0.0368). However, the IgG2b signal against the challenge strain Ngo FA1090 itself did not show a significant interaction, and there was also no difference for any of the isotypes in protected versus not protected 4CMenB animals (Fig. S5).

Considering that 4CMenB is a multicomponent vaccine, we performed western blots with terminal immune (post-immunization and challenge) serum against whole bacterial lysates of both Nme NZ98/254 and Ngo FA1090 to examine if animal-to-animal variability exists in which bacterial components are recognized. Several bands that resolved between 25 and 125 kDa were recognized, with noticeable inter-animal variability in both intensity and number of bands (Fig. S6a and b). Intensities of each distinct band against Ngo FA1090 at two exposures were measured and analyzed against the number of colonized days or cumulative CFU for potential correlations (Fig. S6c). A moderate negative correlation with the number of colonized days was observed for a ~85 kDa (*P* = 0.0220) and a ~65 kDa band (*P* = 0.0196); however, there was no significant difference in intensities between protected versus not protected animals (Fig. S6d and e).

Finally, serum bactericidal activity (SBA) assays were performed against Ngo FA1090 using heat-inactivated 2-fold serially diluted terminal serum and either 2.5% baby rabbit serum (Fig. S7a and b) or 15% normal human serum (Fig. S7c and d) as the complement source. Only 5 of 32 samples produced an SBA titer (dilution at which 50% killing occurs relative to no antibody control) with human serum, to which FA1090 is highly resistant (16), whereas 29 of 32 samples produced an SBA titer using baby rabbit serum. In both assays, there was no significant difference in CFU recovered at each dilution or in SBA titer among adjuvant control and 4CMenB-immunized groups (Fig. S7).

Taken together, these studies suggest high levels of animal-to-animal variability in the humoral response elicited by 4CMenB. Although none of our humoral readouts correlated with protection status, a few promising correlations with the number of days colonized were noted (western blot bands 3 and 6; Fig. S6d and e).

### T cells and CD4+ memory cells increase in 4CMenB-vaccinated mice

**Fig 2:**
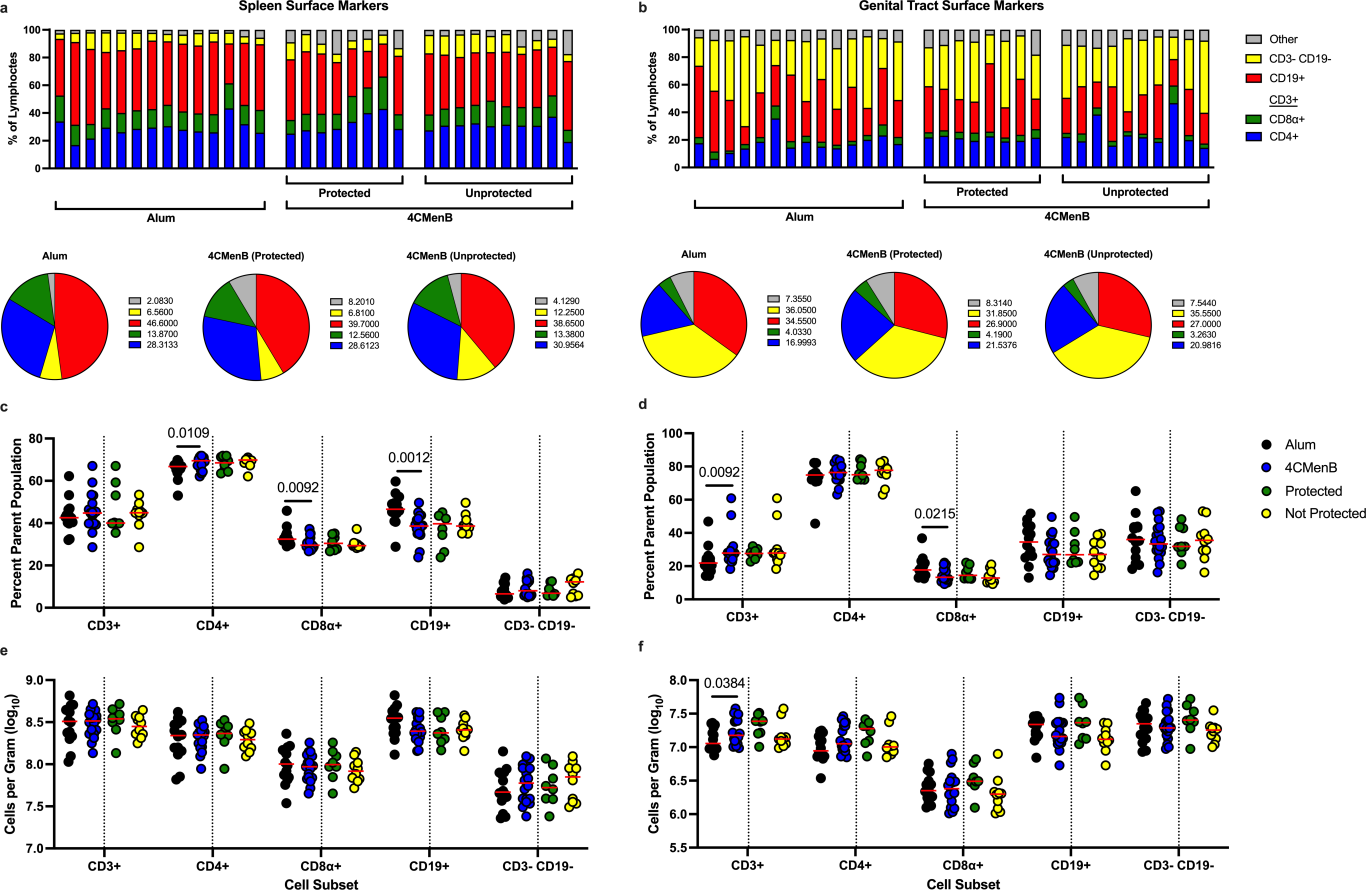
Splenic CD4+ T cell frequencies and genital tract CD3+ T cells increase after immunization with 4CMenB. After three vaccinations of 4CMenB or alum and 6 days post Ngo vaginal challenge, female BALB/c mice were euthanized and necropsied, and frequencies plus total cell counts per gram of tissue were assessed in the spleen and the genital tract using flow cytometry. The frequency of indicated cell subsets is shown in the spleen (a, top) or the genital tract (b, top), with animals divided into their vaccination group and 4CMenB-protection status. Each bar represents one animal. Pie charts denote the median values of each cell subset within each vaccine group in the spleen (a, bottom) or the genital tract (b, bottom). Red, B cells; green, CD8α + T cells; blue, CD4 +T cells; yellow, CD3− and CD19− lymphocytes; gray, remaining live CD45+ cell. Frequency (**c, d**) or absolute count (**e, f**) of each indicated cell subset in the spleen (**c, e**) or genital tract (**d, f**). Each symbol represents one animal, and the red horizontal bar indicates the median. A dotted vertical line separates the two vaccine groups (alum, black; 4CMenB, blue) from the split of the 4CMenB group into protected (green) and not protected (yellow) subsets. *P* values are shown and were generated via Mann-Whitney analysis.

On day 6 post-challenge, mice were euthanized, necropsied, and immune cells were isolated from both the spleen and female genital tract (FGT) and subjected to flow cytometric analysis (Fig. S8) to determine if 4CMenB vaccination had an impact on specific immune cell subsets, and particularly whether the protected and unprotected groups differed. B cells (CD19+), T cells (CD3+), T cell subsets (CD4+ and CD8α+), and non-T non-B lymphocytes (CD3− and CD19−) were assessed (Fig. 2a and b). We noticed a statistically significant increase in CD4+ T cell frequencies in the spleen in our 4CMenB-vaccinated group (*P* = 0.0109), with subsequent decreases in CD8α+ (*P* = 0.0092) and CD19+ cells (*P* = 0.0012; Fig. 2c). In the genital tract, we noted an increase in overall CD3+ T cell frequencies in the 4CMenB group (*P* = 0.0092), with a decrease in the CD8α+ T cells (*P* = 0.0215; Fig. 2d). There were no differences in total cell numbers per gram of tissue in the spleen (Fig. 2e), whereas there were statistically more CD3 +T cells (*P* = 0.0384) in the FGT (Fig. 2f); this difference did not differentiate between protected and unprotected animals.

**Fig 3:**
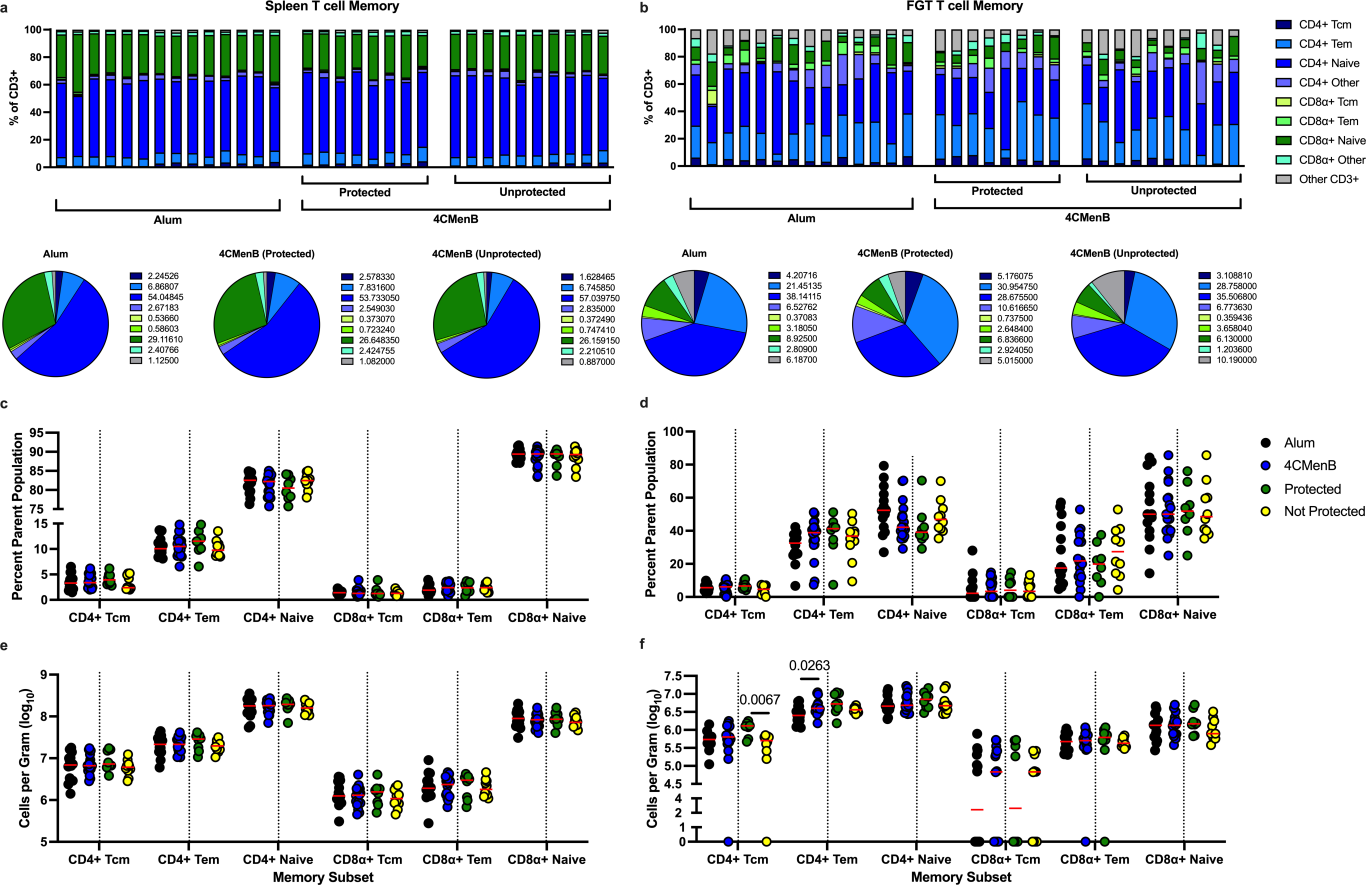
CD4+ T effector memory cell total numbers are increased in the genital tract in 4CMenB vaccinated animals after challenge. After three vaccinations of 4CMenB or alum and 6 days post Ngo vaginal challenge, female BALB/c mice were euthanized and necropsied, and CD4+ and CD8α+ memory cell frequencies plus total counts per gram of tissue were assessed in the spleen and the female genital tract (FGT) using flow cytometry. The frequency of indicated cell subsets is shown in the spleen (a, top) or the genital tract (b, top), with animals divided into their vaccination group and 4CMenB-protection status. Each bar represents one animal. Pie charts denote the median values of each cell subset within each vaccine group in the spleen (a, bottom) or the genital tract (b, bottom). Dark blue, CD4+ T central memory cells (Tcm; CD44+ CD62L+); light blue, CD4+ T effector memory cells (Tem; CD44+ CD62L−); blue, CD4+ naïve cells (CD44− CD62L+); light purple, all other CD4+ T cells (CD44− CD62L−); lime green, CD8α + Tcm (CD44+ CD62L+); green, CD8α + Tem (CD44+ CD62L−); dark green, CD8α + naïve cells (CD44− CD62L+); light green, all other CD8α + cells (CD44− CD62L−); gray, all other CD3 +cells. Frequency (**c, d**) or absolute count (**e, f**) of each indicated cell subset in the spleen (**c, e**) or genital tract (**d, f**). Each symbol represents one animal, red horizontal bar indicates the median. A dotted vertical line separates the two vaccine groups (alum, black; 4CMenB, blue) from the split of the 4CMenB group into protected (green) and not protected (yellow) subsets. *P* values are shown and were generated by using Mann-Whitney analysis.

We also assessed T cell memory subsets in both tissues to determine if 4CMenB vaccination impacted central or effector memory cells (Fig. 3a and b). No frequency differences were noted in the spleen (Fig. 3c); however, there was a trend of increasing CD4+ T effector memory (Tem) cells with a corresponding trend toward decreased CD4+ naive cells in the FGT (Fig. 3d). There were no differences in the total number of cells per gram in the spleen (Fig. 3e), but a significantly higher (*P* = 0.0263) number of CD4+ Tem in the FGT (Fig. 3f). We also noted that there were significantly more (*P* = 0.0067) vaginal CD4+ T central memory (Tcm) cells per gram in protected 4CMenB animals than there were in 4CMenB immunized animals that were not protected (Fig. 3f).

### CD3− CD19− lymphocytes drive a primarily T_H_1-specific immune response in 4CMenB-vaccinated mouse splenocytes

**Fig 4:**
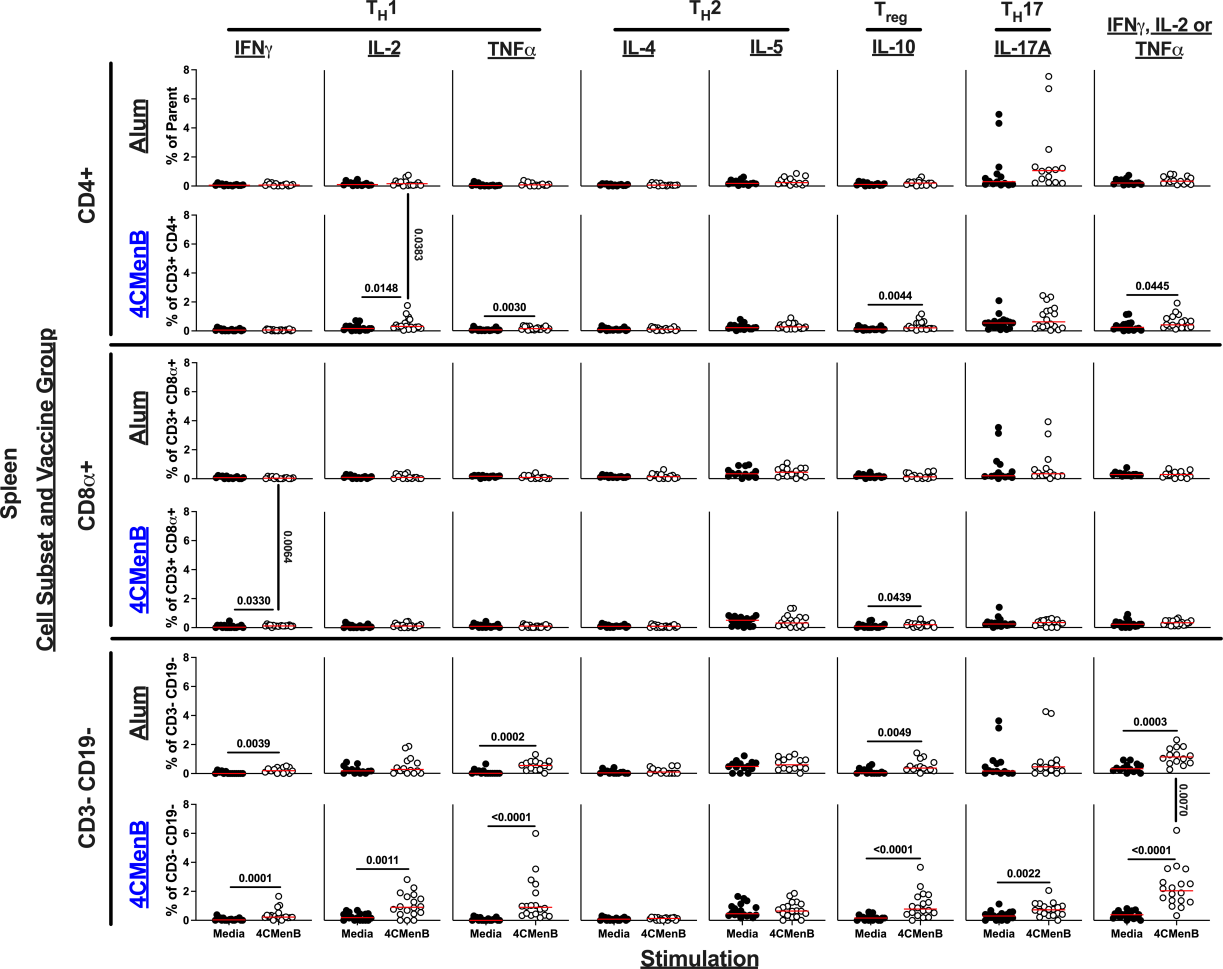
CD3− CD19− splenocytes generate a robust T_H_1 immune response in 4CMenB-vaccinated mice. Splenocytes from necropsied animals on day 6 post-challenge were stimulated to assess the vaccine-specific immune response, which was measured using intracellular cytokine staining and flow cytometry. Each column is the frequency measurement of a specific cytokine (or a combination of any of the T_H_1 cytokines [IFNγ, IL-2, or TNFα as performed by Boolean gating] in the far-right column) after cells were stimulated with either media alone (black) or with 4CMenB (white). Rows indicate both which vaccine group is being assessed (black, alum; blue, 4CMenB) and which cell subset (CD4+, CD8+, or CD3− CD19− lymphocytes). Each symbol is an animal, and the red horizontal bar represents the median of each data set. Non-parametric (Mann-Whitney) analysis was performed both within the vaccine groups (between media and 4CMenB stimulation) and between groups (the alum or 4CMenB stimulated cells compared between alum and 4CMenB vaccinated animals). Significant *P* values are listed with a horizontal black bar if it was within groups or a vertical black bar if it was between vaccine groups.

To uncover the vaccine-specific cytokine response generated by 4CMenB immunization, splenocytes from necropsied animals were stimulated with either cell culture media alone (control) or 4CMenB in the presence of co-stimulatory antibodies (anti-CD49d and anti-CD28) and a cytokine export blocking agent (brefeldin A); hence, individual cell responses could be measured using flow cytometry (Fig. S8). Here, adaptive responses would be considered biologically relevant if there is a difference in the media and 4CMenB-stimulated samples from the 4CMenB-immunized mice group and a difference in the 4CMenB-stimulated samples between alum and 4CMenB-immunized animals. Beyond this, the flow data sets in Fig. 4; Fig. S9 to 12 also highlight numerous non-vaccination-related effects of 4CMenB stimulation, which included significant differences between media-stimulated and 4CMenB-stimulated splenocytes from alum control animals; these presumably result from exposure to endotoxin and/or other bacterial-derived inflammatory mediators present in the vaccine.

Cytokines spanning numerous lymphocyte responses were analyzed including T_H_1 (IFNγ, IL-2, and TNFα), T_H_2 (IL-4 and IL-5), T regulatory (T_reg_, IL-10), and T_H_17 (IL-17A) in both conventional (CD4+ and CD8α+) and unconventional (CD3− CD19−) cell subsets (Fig. 4). Our experiment demonstrated that 4CMenB vaccination elicits a significant increase in the frequency of CD4+ IL-2+ cells (*P* = 0.0148, media vs. 4CMenB stimulation, 4CMenB-vaccinated animals; *P* = 0.0383, alum vs. 4CMenB vaccinated animals, 4CMenB stimulation) and CD8α+ IFNγ+ cells (*P* = 0.0330, media vs. 4CMenB stimulation, 4CMenB-vaccinated animals; *P* = 0.0064, alum vs. 4CMenB vaccinated animals, 4CMenB stimulation). Given the importance of cells producing multiple cytokines in vaccine responses (17), we also performed Boolean gating to capture cells producing any combination of T_H_1 cytokines (IL-2, IFNγ, and TNFα). Unexpectedly, we observed a statistically significant increase in the frequency of CD3− CD19− cells producing any T_H_1 cytokine (*P* < 0.0001, media vs. 4CMenB stimulation, 4CMenB-vaccinated animals; *P* = 0.0070, alum vs. 4CMenB vaccinated animals, 4CMenB stimulation). When analyzing the responses on a number of cells per gram basis, we did not see any significantly different 4CMenB-mediated immune responses, although CD3− CD19− TNFα+ cells (*P* < 0.0001, media vs. 4CMenB stimulation, 4CMenB-vaccinated animals; *P* = 0.0504, alum vs. 4CMenB vaccinated animals, 4CMenB stimulation) and CD3− CD19− cells producing any T_H_1 cytokine (*P* = 0.0002, media vs. 4CMenB stimulation, 4CMenB-vaccinated animals; *P* = 0.0504, alum vs. 4CMenB vaccinated animals, 4CMenB stimulation) were approaching significance (*P* = 0.0504; Fig. S9). Comparing 4CMenB vaccinated animals based on whether they were protected also elucidated that not protected animals had a higher percentage of CD8+ cells that produced IL-10 (*P* = 0.0029, media vs. 4CMenB stimulation, 4CMenB-vaccinated not protected animals; *P* = 0.0230, not protected vs. protected 4CMenB vaccinated animals, 4CMenB stimulation) and protected animals had a higher percentage of CD3− CD19− cells producing TNFα (*P* = 0.0007, media vs. 4CMenB stimulation, 4CMenB-vaccinated protected animals; *P* = 0.0263, protected vs. not protected 4CMenB vaccinated animals, 4CMenB stimulation) (Fig. S10). These significant differences were not present when evaluating the total number of cells per gram of tissue (Fig. S11). Delving into CD3− CD19− cells producing different combinations of T_H_1 cytokines further, we noted that 4CMenB-vaccinated mice had statistically higher CD3− CD19− cells producing IL-2 and TNFα only compared with control animals based both on a percent-parent population (*P* = 0.0136) and a total cell per gram basis (*P* = 0.0190) (Fig. S12a). Importantly, when comparing only protected from not protected 4CMenB-vaccinated animals, these statistical differences held on a percent-parent population (*P* = 0.0311), and we noted a significant difference in CD3− CD19− cells producing only IFNγ and TNFα on both a percent parent (*P* = 0.0257) and total cell per gram basis (*P* = 0.0156), suggesting that these cellular phenotypes (robust production of TNFα with either IL-2 or IFNγ by antigen-stimulated CD3− CD19− cells) may serve as important performance indicators (Fig. S12b).

### Multiplex analysis demonstrates a robust cytokine response to 4CMenB immunization

**Fig 5:**
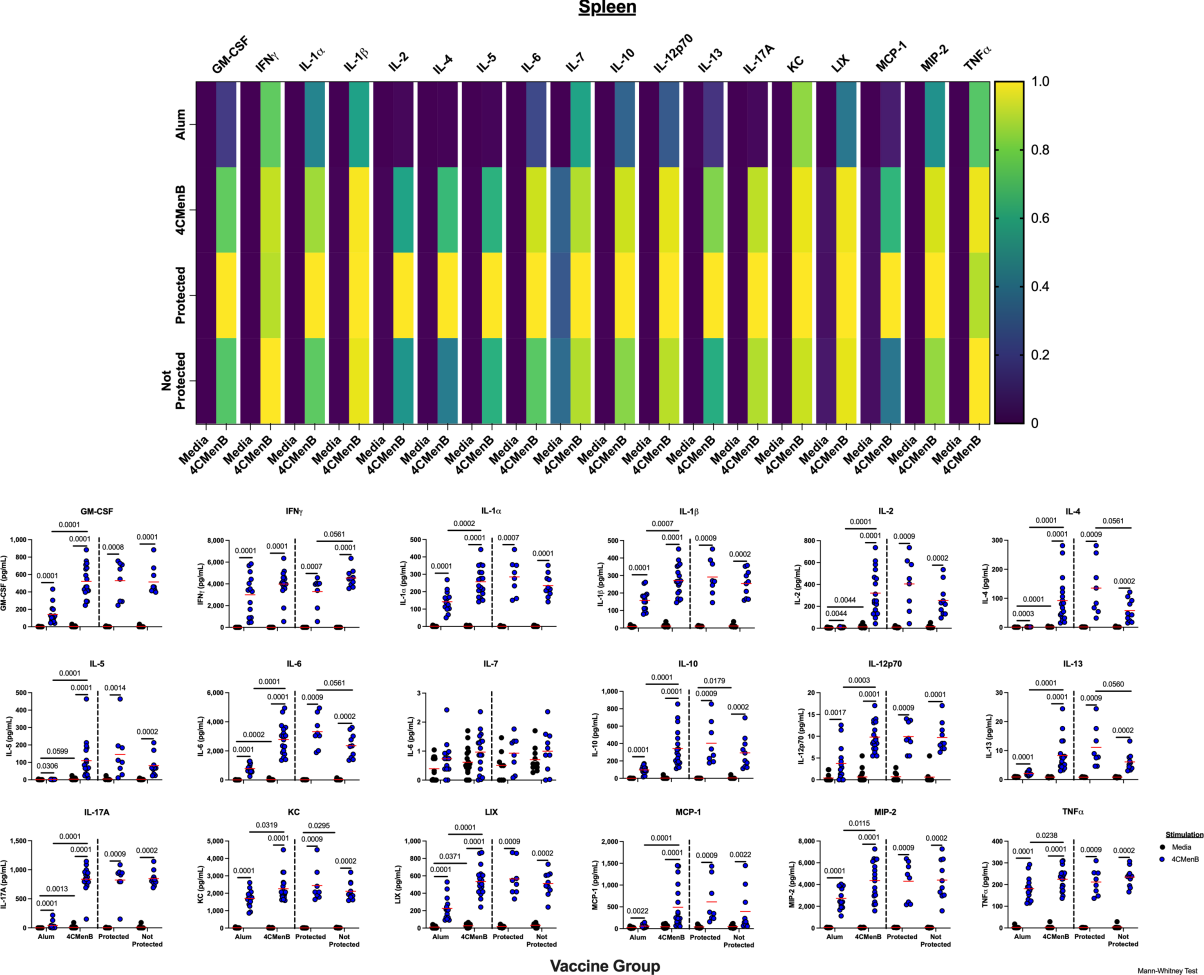
4CMenB vaccinated splenocytes secrete a multitude of cytokines from across the T cell subtype spectrum in response to 4CMenB stimulation. Splenocytes from vaccinated and infected animals were incubated with either media alone or 4CMenB, and the secreted cytokine response from the culture media was measured using a T cell-centric 18-plex. Top, a heatmap of normalized values for each cytokine listed along the top from when cells were stimulated with either media or 4CMenB (columns) in the indicated group (top row, alum vaccinated animals; second row, all 4CMenB vaccinated animals; third row, only protected 4CMenB animals; bottom row, not protected 4CMenB vaccinated animals). Bottom, scatterplots of the raw cytokine data used to generate the heat map. Each cytokine is listed with each symbol representing the measure of an individual animal’s splenocytes after stimulation with either media (black) or 4CMenB (blue). Indicated groups are listed on the *X*-axis. Red horizontal lines indicated the median values, and black horizontal lines connected two compared groups with statistically different medians (using Mann-Whitney non-parametric comparison) with *P* values listed above.

For a broader assessment of the vaccine-specific cytokine responses generated by 4CMenB immunization, lymphocytes from the spleen and the FGT were stimulated with either 4CMenB or cell culture media alone, and secreted cytokines were then measured by a T cell-focused 18-plex assay (Fig. 5; Fig. S13). Cytokine responses from the spleen showed a multi-faceted 4CMenB-specific response, with significantly higher amounts of T_H_1 (IL-2, TNFα, IL-12p40), T_H_2 (IL-4, IL-5, IL-13), T_reg_ (IL-10), T_H_17 (IL-17A), more broadly associated pro-inflammatory cytokines (IL-1α, IL-1β, and IL-6), and other important cytokines/chemokines (GM-CSF, KC, LIX, MCP-1, and MIP-2). Notably, T_H_2 (IL-4 and IL-13) and IL-6 were significantly elevated in the 4CMenB-vaccinated protected group compared with the not protected group. Significant vaccine-specific responses in the FGT were more limited and included T_H_1 (IL-2), T_H_2 (IL-4), and T_H_17 (IL-17A). In both data sets, there were also numerous non-memory, inflammatory-related effects of 4CMenB stimulation (Fig. 5; Fig. S13).

### Cytokines and chemokines in the vaginal lumen decrease as gonococci are cleared in 4CMenB-vaccinated animals

To better understand the environment in the vaginal lumen prior to and throughout gonococcal infection, we assessed 45 different analytes prior to infection, as well as on days 3 and 6 post-challenge. The only cytokine that was different between vaccine groups prior to infection was a statistically reduced concentration of the tissue inhibitor of metallopeptidases (TIMP-1) in the vaginal lumen of 4CMenB-vaccinated animals (*P* = 0.0262; Fig. S14). On day 3 post-challenge, 4CMenB-vaccinated protected animals had statistically lower IL-7 and erythropoietin (EPO), and some animals displayed higher IL-1β compared with animals that were not protected (*P* = 0.0453, 0.0354, and 0.0183, respectively; Fig. S14). Finally, on day 6, we saw a reduction in twelve different analytes in 4CMenB-vaccinated animals compared with alum vaccinated animals, including chemokines (eotaxin, fractalkine, MCP-5, MDC, and TARC), growth factors (G-CSF and M-CSF), pro-inflammatory cytokines (IL-1β, IL-6, IL-12p40, and IL-16), and one cell differentiation inhibitor (LIF), whereas the only difference in the protected versus not protected 4CMenB vaccinated group was less EPO in the protected group.

### Principal component analyses of complex data sets

To consider together the variability in responses among the multiple complex data sets described above, principal component analysis (PCA) was performed. For the 18-plex T cell cytokine data set from 4CMenB-stimulated splenocytes, 80.7% of the variability could be attributed to the first three components (Fig. S15a). The resulting loading matrix shows that the first principal component (PC1) is positively correlated with many of the measured analytes (Fig. S15b). This implies PC1 is a multifaceted measurement, driven by the production of T_H_1 (IL-2, IL-12p70), T_H_2 (IL-4, IL-5, IL-13), T_H_17 (IL-17A), T_reg_ (IL-10), and other potent cytokines and chemokines (GM-CSF, IL-1α, IL-1β, LIX, MCP-1, and KC). PC2 increased with T_H_1 cytokines (TNFα and IFNγ) and decreased with T_H_2 cytokines (IL-5 and IL-13). PC3 was primarily driven by a positive correlation with IL-7, a growth factor that stimulates T, B, and NK cells. Scores for PC1, PC2, and PC3 were compared between the vaccine groups, and it was shown the animals given 4CMenB had a higher mean PC1 score compared with animals given alum (*P* < 0.0001; Fig. S15c), whereas PC2 and PC3 showed no difference between the groups (Fig. S15d and e).

We also performed PCA on the flow cytometric frequencies (Fig. S16) and total cell counts per gram of tissue (Fig. S17) of 4CMenB-stimulated splenocytes, as well as on the 45-plex cytokine data from the vaginal lavage of animals pre- and post-challenge (Fig. S18). Notable highlights from these analyses included significantly higher scores in principal components from 4CMenB-vaccinated animals comprised of positively correlated frequencies and total cells of CD3− CD19− cells secreting TNFα alone or in combination with other T_H_1 cytokines (Fig. S16d and S17e, respectively). We also observed a significantly lower score in a total cell principal component that was highly correlated with total CD4+ T central memory and CD8α+ cells producing cytokines (Fig. S17d). 4CMenB vaccinated animals had a statistically lower score in a day 6 post-challenge vaginal lavage component that was highly correlated with a large number of chemokines and pro-inflammatory cytokines (Fig. S18, bottom), whereas protected 4CMenB immunized animals had a lower average score compared with not protected animals in a pre-infection lavage component that was positively correlated with certain chemokines (fractalkine and KC) and negatively correlated with others (TARC, 6Ckine/Exodus 2, and IP-10) (Fig. S18, top). These findings both support our previous results of the importance of CD3− CD19− cells that produce TNFα and other cytokines and provide novel directions to explore vaginal cytokine and chemokine responses.

## DISCUSSION

The serogroup B meningococcal vaccine 4CMenB has been shown to elicit modest but potentially impactful cross-protection against Ngo infection (12), raising exciting questions regarding both what cross-protective antigens are targeted by this multi-valent vaccine and what immune mechanism(s) are responsible for protection against Ngo. In considering the latter question, we describe here a comprehensive immune analysis to evaluate the humoral and cellular immune responses elicited by 4CMenB upon gonococcal challenge in mice. Extending upon prior work that considered protection and the humoral response elicited by 4CMenB in mice (15), we now provide the first comprehensive analysis of the cellular immune response elicited by this vaccine and consider together the cellular and humoral responses in mice in the context of protected or not protected infection outcomes. We observed that immunization with 4CMenB elicited a significant reduction in the duration of vaginal colonization and a decrease in bacterial load of Ngo in a subset of mice, reflecting the efficacy apparent in retrospective studies in humans who have received 4CMenB immunization for protection against Nme. Similar efficacy against Ngo in the murine lower genital tract infection model has been reported by others (15), which reinforces the robustness of this cross-protective response. A significant strength of our study design was the use of pilot data to properly power the main study. This allowed us to adopt a rigorous statistical framework for defining potential outcomes and correlates while also allowing us capacity to broadly evaluate serology, cellular immune responses, and cytokine responses.

Prior studies have established that 4CMenB immunization is able to elicit cross-reactive antibodies that bind multiple gonococcal surface components and, in some cases, also facilitate complement-mediated lysis of gonococci (7, 8, 15). We also found that vaccination with 4CMenB-induced serum IgG antibodies that bound both meningococci and gonococci. We extended beyond the prior reports by dissecting the specific IgG isotypes that were binding to each antigen, as well as examining if there is animal-to-animal variability in the components being recognized. A negative correlation between the number of colonized days and cumulative CFU with IgG2b specific for Nme NZ98/254 was an unexpected finding and is provocative considering the relatively low IgG2b signals detected against Ngo FA1090 itself. However, the negative correlation between the number of colonized days and two anti-FA1090 proteins of approximately 85 kDa and 65 kDa was particularly interesting, given that these correspond to the sizes of the outer membrane proteins BamA and NHBA, respectively. This highlights the utility of using such a more granular approach to determine antibody correlates due to the complexity of 4CMenB’s antigen composition. From a mechanistic point of view, the contribution of these antibodies during clearance in our model remains unclear since we were unable to detect anti-gonococcal antibodies in the vaginal lumen of vaccinated animals (Fig. S3). This could either be due to our route of immunization being subcutaneous, which others have found to not elicit significant anti-gonococcal antibodies in the vagina (15) or due to our measurements being performed on post-challenge terminal lavages in which anti-gonococcal antibodies may have been specifically depleted by the infecting bacteria.

The cellular immune response we detected in our animal cohorts was revealing but highly complex. From an overall immune cell subset perspective, we observed a statistically significant increase in CD4+ T cell frequencies in the spleen and CD3+ T cells and CD4+ T effector memory cells in the genital tract of vaccinated animals (Fig. 2 and 3). Although the magnitude of these responses was modest, it is important to note that our experiment was not designed to analyze changes occurring within antigen-specific T cell subsets specifically. Therefore, this nuanced change in overall T cell population is presumably the result of a more robust response within the antigen-specific T cell subpopulations. Also worth noting is that because the measurements can only be monitored at the experimental endpoint, this study provides a snapshot into vaccination-induced changes in lymphocyte and cytokine responses at a single post-challenge time point, with factors such as current bacterial loads and duration of colonization contributing to the local immune dynamics in each individual.

The vaccine-induced cytokine response noted here was extremely robust. Multiplex ELISA data showed elevated levels of cytokines that are associated with many prominent immune subsets (T_H_1, T_H_2, T_reg_, and T_H_17) when splenocytes harvested from 4CMenB-vaccinated animals were re-stimulated. Indeed, our PCA analysis indicated that over 57% of variability in this data set was due to one principal component that was positively correlated with many of these cytokines/chemokines, and mean scores were statistically higher in 4CMenB-vaccinated animals. Many of the cytokines we observed in our study closely resemble those seen from human PBMCs co-cultured with gonococci (18), reinforcing the validity of using animal models for studying the immune response to *Neisseria*. We also noted that some of the cytokines that we observed to be significantly higher in 4CMenB-vaccinated animals have been previously suggested to be important in the control of gonococcal infection, including IL-17 (19) and T_H_1 (20, 21) responses. Although we did not find any single cytokine response to correlate with protection, it is possible that a robust, multifaceted response is what is required to protect against Ngo, and future studies should take this into account.

The flow cytometric analysis of stimulated splenocytes indicated that although CD4+ and CD8+ T cells from 4CMenB-immunized animals contribute to the production of T_H_1 (IFNγ, IL-2, and TNFα) and T_reg_ (IL-10)-associated cytokines, by far the most noteworthy response was coming from the non-T non-B lymphocytes. These cells were producing primarily T_H_1 cytokines (IFNγ, IL-2, and TNFα), and Boolean gating showed the main difference between both the alum and 4CMenB group as well as protected and not protected 4CMenB vaccinated animals was a subset of these cells producing TNFα in combination with either IL-2 or IFNγ (Fig. S12). Non-T non-B lymphocytes in this study were defined by a negative gating strategy in that they expressed neither CD3 (the T cell receptor) nor CD19 (a B cell lineage marker), which begs the question of what exactly they are? The most likely answer is NK cells or a member of the innate lymphocyte cell (ILC) lineage 1 family, given their anatomical location (22, 23) and the strong T_H_1 response observed (24, 25). The role of both of these cell subsets in gonococcal infection is largely unknown (26); however, other studies have shown that vaccines can stimulate these cell subsets, and they can be critical for protection in other infection models (27, 28). Since T cells that produce more than one cytokine are important in vaccine studies (17, 29), future analyses should aim to identify this cell population and consider its ability to promote gonococcal clearance from the female genital tract.

Examining the local immune responses within the genital tract offered further insights into the complex and dynamic interplay of cytokines and chemokines over the course of infection. The unbiased analysis of 45 different cytokines on day 6 post-challenge of vaginal lavages showed 4CMenB-vaccinated animals having lower concentrations of numerous pro-inflammatory cytokines/chemokines, including IL-6, IL-12p40, IL-16, eotaxin, MCP-5, and fractalkine. This finding was also supported in PCA analysis of this data set whereby 4CMenB-vaccinated animals had statistically lower mean scores of a component that explained nearly 40% of the variance in this data set and was comprised of many of the factors listed above. This appears to reflect the waning response as 4CMenB-vaccinated animals clear their infection, whereas control animals would still be actively inflamed from the presence of the gonococci. Assessing vaginal lavages prior to infection was more interesting. Although only TIMP-1 was statistically lower in 4CMenB-vaccinated animals compared with our controls from a concentration perspective, PCA analysis indicated that protected 4CMenB-vaccinated animals had statistically lower mean scores in one component, which described over 10% of the variance and was negatively correlated with a few key T cell/NK cell chemoattractants (TARC, 6Ckine/Exodus 2, and IP-10) (30–32). This, along with the higher T effector memory cell counts observed in the genital tract of 4CMenB-immunized animals, supports the premise that T cells are recruited and/or proliferate in the FGT during infection. *Ex vivo* stimulation of lymphocytes derived from the genital tract further supports that vaccinated animals are capable of producing significantly greater levels of cytokines upon antigen encounter, including cytokines that promote T cell proliferation and survival (IL-2), B cell differentiation (IL-4), and mediate proinflammatory responses (IL-17), of which IL-17 has been previously linked to gonococcal clearance (19; reviewed by Raphael et al. [33]).

The female mouse lower genital tract infection model used in this study is now widely accepted for preclinical evaluation of vaccines and antibiotics against gonorrhea; however, we remain mindful of its limitations. First and foremost, the gonococci are highly adapted to life in humans; hence, virulence factors involved in processes such as cellular adherence and nutrient acquisition are not considered in wild-type mice. Although humanized transgenic mice are available to allow these factors to be considered (34, 35), the complexities associated with breeding these animals are restrictive in studies where a large cohort of female mice of the same age is required. Considering the female genital tract itself, hormonal fluctuations can influence the immune response and susceptibility to infection (36). Given that mice in diestrus tend to resist gonococcal infection (14), it remains unknown how the response to infection would be affected during progesterone-dominant stages of reproductive cycling. It must also be noted that because mice must be in the correct estrous stage prior to infection, we often must infect different cohorts of mice over many days for one experiment. Preparing a daily inoculum of Ngo introduces variability in the infection doses. In this regard, it is interesting that we did observe protection in a few of the 4CMenB-vaccinated animals that were administered the lowest doses of *Ngo* (≤3 × 10^6^ CFU). These mice were infected, evident by the fact that Ngo was recovered from vaginal lavages early after infection, a dose-dependent impact on protection cannot be excluded; hence, future experiments should explore this. It is also worth mentioning that for our *ex vivo* antigen-stimulation experiments, we used 4CMenB as the stimulant in efforts to achieve the maximum response. Although gonococci and meningococci share substantial homology (37), some key components in 4CMenB such as PorA, which is a major component of the OMV, fHbp, NadA, and both fusion proteins (GNA1030 and GNA2091) are not present/surface exposed in *N. gonorrhoeae*. Thus, OMVs from diverse gonococcal isolates would provide a more accurate measure of the strain-specific diversity of antigen-driven cellular responses for future correlation studies.

In summary, we have established that a robust cellular response develops following 4CMenB immunization, with T effector cells and unconventional lymphocytes (non-T non-B) appearing to play a central potential role in driving gonococcal clearance. Our findings highlight that focusing solely on antibody titers and serum bactericidal assays may not reveal the mechanistic determinants of protection and that a comprehensive analysis that integrates the humoral and cellular immune responses will likely be required to define the correlates of protection. As multiple clinical trials are ongoing with 4CMenB, and others are being implemented to test other vaccine formulations, we hope that insights gained from this study will benefit future vaccine assessments.

## MATERIALS AND METHODS

### Animals

Mice used in this study were 6- to 8-week-old BALB/c females (Charles River). Upon reception to the University of Toronto Division of Comparative Medicine (DCM) animal facility, animals were acclimatized to the environment for at least 7 days before any handling was performed. One pilot experiment and two independent vaccination and challenge studies were conducted for this study. A list of experimental animals and corresponding information can be found in Table S1.

### Vaccination

4CMenB (Bexsero, GSK) vaccine was purchased at a pharmacy and stored at 4°C until use. Mice were subcutaneously (s.c.) vaccinated with either a one-half human dose (0.25 mL) or an equivalent amount of aluminum hydroxide adjuvant (0.25 mg) plus phosphate-buffered saline (PBS; Wisent Bioproducts, Canada). Vaccinations occurred every 3 weeks for a total of three vaccinations, with a 2-week rest period before animals were staged into infection.

### Staging and preparation of mice

To determine the stage of the reproductive cycle, mouse vaginas were lavaged daily with 30 µL of PBS using a 100 µL micropipette. Wet smears were examined microscopically under a 40× objective lens, and the stage of the estrous cycle was determined based on cytology (38). Mice transitioning from estrus or metestrus to diestrus over a 24 h timespan were considered cycling normally and included in the study. Upon confirmation of proper cycling, mice were s.c. injected with 200 µL water-soluble β-estradiol (0.5 mg/mouse; Sigma-Aldrich, Canada) to induce estrus 2 days prior to infection. Mice were subsequently injected with β-estradiol on the day of infection and day 2 post-challenge to sequester the mice in this stage. The cycling mice also received intraperitoneal (i.p.) injections of vancomycin (2.4 mg) and streptomycin (0.6 mg) in 200 µL of PBS for 4 days (day −2 to day +1 relative to infection). During the same time span, mice also received trimethoprim antibiotics in their drinking water at a final concentration of 0.4 mg mL^−1^. On day 2 post-challenge, the antibiotics in the water were changed to additionally contain streptomycin sulphate (2.4 mg mL^−1^) in addition to trimethoprim. This regimen continued throughout the entirety of the experiment.

### Preparation of *N. gonorrhoeae* inoculum

*N. gonorrhoeae* strain FA1090 was streaked out onto GC agar (Becton Dickinson, Canada) supplemented with Kellogg’s (D-glucose 4 g/L, glutamine 50 mg/L, ferric nitrate 5 mg/L, and cocarboxylase 0.2 mg/L final concentration) and vancomycin, colistin, nystain and trimethoprim (VCNT; 300 µg vancomycin, 750 µg colistin, 1,200 U nystatin, and 500 µg trimethoprim lactate; Becton Dickinson, Canada) antibiotics and incubated overnight at 37°C plus 5% CO_2_. The overnight lawn of gonococci was harvested into 1 mL of PBS supplemented with 0.9 mM CaCl_2_ and 0.5 mM MgCl_2_ (PBS++; Wisent Bioproducts, Canada), and the optical density at 550 nm (OD_550_) was measured to calculate the desired concentration of bacteria.

### Vaginal infection and bacterial recovery

The natural estrous cycle of the mice was monitored for approximately 5 days, and once proper cycling was confirmed, hormone and antibiotic treatments were initiated as indicated above. On day 0, approximately 1 × 10^7^ colony-forming units (CFU) of mouse-passaged *N. gonorrhoeae* FA1090 bacteria (see Table S1 for exact infection doses for each animal) in 2.5–5 µL PBS++ were inoculated intravaginally on non-sedated mice using a P10 pipette. For gonococcal recovery, the vagina was washed daily with 15 µL of PBS++ and serial dilutions were plated on GC agar supplemented with Kellogg’s and VCNT, which suppresses the growth of commensals while allowing the recovery of *N. gonorrhoeae*. Animals were considered protected if they had no bacterial recovery on at least days 4–6 (therefore fulfilling the cleared criteria above).

### Cell isolation for immunological assays

On day 6 post-challenge, mice were humanely euthanized using 100% CO_2_ exposure. Both the spleen and genital tract were sterilely excised, placed into complete media (cRPMI; RPMI-1640 [Wisent Bioproducts], supplemented with 10% fetal bovine serum, 20 mM HEPES [Gibco], 1% [vol/vol] GlutaMAX [Gibco] 10 units mL^−1^ penicillin and 10 units mL^−1^ streptomycin;) on ice and weighed. Spleens were processed into a single-cell suspension by mechanically pressing tissue through a 40 µM cell strainer. Cells were treated to remove red blood cells via incubation in 1× RBC lysis buffer (eBioscience) for 5 min in the dark at room temperature. The cells were then washed with PBS and resuspended in cRPMI for counting using a hemocytometer. Female genital tracts (FGT) were finely minced using surgical scissors and incubated for 30 min in digestion buffer (Hanks balanced salt solution [HBSS; Gibco] supplemented with 5% FBS, 10 mM HEPES [Gibco], 20 µg mL^−1^ DNase I and 2 mg mL^−1^ collagenase D) at 37°C with shaking. The tissue was then pressed through a 40 µM cell strainer, washed with PBS, and resuspended in cRPMI for counting using a hemocytometer.

### Flow cytometry

One million cells from either the spleen or the FGT were added to the wells of a round-bottom 96-well plate. They were then stimulated with either a solution of 4CMenB (final concentration of 0.5 µg mL^−1^ of the outer membrane vesicle [OMV; measured as amount of total protein]) and anti-CD49d (clone R1-2; final concentration 1 µg mL^−1^) plus anti-CD28 (clone 37–51; final concentration 1 µg mL^−1^) antibodies or cRPMI media with the same antibody cocktail for 2 h at 37°C plus 5% CO_2_. To assess intracellular cytokines, brefeldin A (GolgiPlug, BD Biosciences) was added as per the manufacturer’s instructions, and the cells were left to incubate as indicated previously for an additional 12 h. Treated samples were then transferred to a 96-well V-bottom plate, spun at 500 × *g*, and washed twice with PBS. All the following steps were completed in a low to no light setting. The cells were incubated with a viability stain (BD Horizon Fixable Viability Stain 620; BD Biosciences) for 15 min at room temperature, washed twice with fluorescence activated cell sorting (FACS) buffer (PBS + 1% FBS), and incubated with Fc block (TruStain FcX PLUS [anti-mouse CD16/32], BioLegend) for 10 min at 4°C. The surface stain antibody cocktail was then added, and the samples were incubated for an additional 30 min at 4°C. Cells were washed twice with FACS buffer and resuspended in fixation/permeabilization solution (BD Biosciences) for 20 min at 4°C. The samples were washed twice with Perm/Wash buffer (BD Biosciences), and the intracellular antibody cocktail was added and incubated at 4°C for 30 min. The samples were washed two more times with Perm/Wash buffer, resuspended in FACS buffer, and kept at 4°C in the dark until they were read on a FACSymphony A3 5-Laser Cell Analyzer (BD Biosciences). Flow cytometry data were analyzed using FlowJo version 10.9.0 (Becton Dickinson). A list of all flow cytometry antibodies used in this study can be found in Table S2.

### Cytokine multiplex assays

One million cells from either the spleen or the FGT were added to the wells of a round-bottom 96-well plate and were stimulated with either 4CMenB (final concentration of 0.5 µg mL^−1^ of the OMV [measured as amount of total protein]) or cRPMI media alone for 24 h at 37°C plus 5% CO_2_. The cells were then spun down at 500 × *g*, and the supernatants were stored at −20°C. Murine vaginal lavage samples from 2 weeks post-vaccination (pre-infection) as well as days 3 and 6 post-challenge were spun down, and supernatants were heat treated at 56°C for 30 min (to kill any live *N. gonorrhoeae*) and subsequently stored at −20°C. All samples were shipped on dry ice to Eve Technologies (Calgary, Alberta, Canada) to perform multiplex assays.

### *Neisseria*-specific antibody titer analysis

Nme NZ98/254 and Ngo FA1090 were grown overnight on GC-Kellogg’s at 37°C plus 5% CO_2_. Lawns were resuspended in PBS++ and OD_600_ measured and adjusted to an OD = 0.5 by adding additional PBS++. Bacterial suspensions were heat-inactivated at 57°C for 1 h, and 20 µL/well was used to coat 384-well ELISA plates (MaxiSorp, Non-Sterile, ThermoFisher, cat. 464718) and dried in a biological safety cabinet. Once dried, plates were stored at 4°C until use. Plates were blocked with 5% bovine serum albumin (BSA; BioShop Canada, cat. ALB001) for 2 h at room temperature, washed three times with PBS plus 0.05% Tween (PBST), incubated overnight at 4°C with mouse serum diluted 1:20,000 in 1% BSA or vaginal lavages diluted 1:5 or 1:2 in 1% BSA, washed again three times with PBST, and incubated with secondary detection antibody diluted in 1% BSA for 2 h at room temperature. For isotypes, alkaline phosphatase conjugated goat anti-mouse IgG1, IgG2a, IgG2b, and IgG3 antibodies (Jackson ImmunoResearch, cat. 105-055-205, 105-055-206, 105-055-207, and 105-055-209, respectively) at 1:5,000 dilutions were used, and the plates subsequently washed and developed with BluePhos substrate (Mandel KP 5120-0059) and reads obtained at 620 nm. For isotype comparison, plates were set up to allow for all four isotypes to be quantified for all samples against both Nme and Ngo on a single plate. Reads from three replicate plates were averaged. For total IgG and IgA quantification, goat anti-mouse secondary detection antibody conjugated to HRP was diluted 1:10,000 for IgG (peroxidase conjugated goat anti-mouse IgG H&L, Jackson ImmunoResearch, cat. 115-035-003) and 1:5,000 for IgA (peroxidase conjugated goat anti-mouse IgA, Southern Biotech, cat. 1040-05). Some wells of the same plates were coated with anti-mouse IgG/IgA capture antibody instead of heat-inactivated bacteria, and standards ranging from 125 ng/mL to 1.953 ng/mL were added to these wells at the same time as the primary. ELISAs with peroxidase-conjugated secondary antibodies were developed with KPL SureBlue TMB Microwell Peroxidase Substrate (SeraCare, cat. 5120-0077), the reactions were quenched with 2 N H_2_SO_4_, and readings were obtained at 450/520 nm.

### Western blots

Nme NZ98/254 and Ngo FA1090 were grown overnight on GC-Kellogg’s at 37°C plus 5% CO_2_. Lawns were resuspended in PBS++ and OD_600_ measured and adjusted to 3. Bacterial suspension was added to 2× sodium dodecyl sulfate (SDS) buffer (0.125 M Tris, 4% [wt/vol] SDS, 20% [vol/vol] glycerol, and 0.01% [wt/vol] bromophenol blue) at a ratio of 1:2, boiled for 10 min and then stored at −20°C until use. 2-mercaptoethanol was added to the sample at a final concentration of 5%, 25 µL sample/well was resolved on 10% SDS-PAGE. After transfer, PVDF membranes were blocked in 5% skim milk in Tris-buffered saline with 0.05% Tween (TBST) for 2 h at room temperature and then incubated overnight at 4°C with 1:5,000 dilution of terminal mouse serum in 1% skim milk. After 3 TBST washes, peroxidase conjugated goat anti-mouse IgG H + L (Jackson Immunoresearch, cat no. 115-035-003) was used at 1:20,000 dilution in 1% skim milk for 2 h at room temperature, washed three times with TBST and developed with the Novex ECL Chemiluminescent Substrate Reagent Kit (Invitrogen). Images were obtained with an Invitrogen iBright CL750 Imaging System and densitometry analysis performed on ImageJ software (v1.54g).

### Serum bactericidal assay

Ngo FA1090 was grown overnight on GC-Kellogg’s agar at 37°C plus 5% CO_2_, subcultured onto a new GC-Kellogg plate, and grown for an additional 4.5 h in the same conditions to obtain a lawn of logarithmic phase growth organisms. Bacteria were then resuspended in RPMI, OD_550_ measured, and ~250–1,000 CFU were added to heat-inactivated mouse sera serially diluted in RPMI in 96 well-round bottomed plates. After 5 min, either pooled normal human serum (Sigma, cat. H4522, Lot #SLCD1946) or baby rabbit complement (Cedarlane, CL3441-S100) at a final concentration of 15% and 2.5%, respectively, in a total reaction volume of 40 µL. After 60 min of incubation at 37°C plus 5% CO_2_, 10 µL was plated on GC-Kellogg’s agar. Serum bactericidal assay (SBA) titers were reported as the highest dilution at which there was >50% killing compared with complement source alone.

### Principal component analysis of cellular, cytokine, and chemokine parameters

Principal component analysis (PCA) was performed on multi-dimensional data in JMP Pro version 17.2.0 (SAS Institute Inc., Cary, NC). For 18-plex T cell cytokine/chemokine data, PCA was run on 4CMenB-stimulated splenocytes only. For flow cytometry data, PCA was run on both frequencies and counts from 4CMenB-stimulated splenocytes. For vaginal lavage 45-plex data, PCA was run separately at each time point. PCA was performed on correlations (of centered and standardized variables) using row-wise variance estimation. Loading matrices for the first three principal components are included in supplemental material (Tables S3 and S8).

### Statistical analysis and graphing

Power calculations were based on the percent of colonized animals in a pilot study. The sample size was determined to be a minimum of 14 animals per group, providing 80% power to detect a statistical difference in colonization at day 6 with an alpha of 0.05.

All statistical analyses and figure creation were performed using either JMP Pro version 17.2.0 or Prism version 10.2.3 (GraphPad Software LLC., La Jolla, CA).

## Data Availability

The information on each animal (unique identifier, treatment group, infection cohort/bacterial dose, and bacterial enumeration throughout study) can be found in Table S1. Loading matrices for all PCA plots can be found in Tables S3 to S8. Raw data for antibody titers, flow cytometry cell subset percentages, absolute counts, flow plots, and multiplex data sets are available upon request.

## SUPPLEMENTAL MATERIAL

Supplemental figures, part 1 msphere.00940-24-s0001.pdf Fig. S1 to S6.

Supplemental figures, part 2 msphere.00940-24-s0002.pdf Fig. S7 to S12.

Supplemental figures, part 3 msphere.00940-24-s0003.pdf Fig. S13 to S18.

Supplemental tables msphere.00940-24-s0004.xlsx Tables S1 to S8.

ASM does not own the copyrights to Supplemental Material that may be linked to, or accessed through, an article. The authors have granted ASM a non-exclusive, world-wide license to publish the Supplemental Material files. Please contact the corresponding author directly for reuse.
