# Supplementary material for: Meningococcal vaccine 4CMenB elicits a robust cellular immune response that targets but is not consistently protective against Neisseria gonorrhoeae during murine vaginal infection
Source: mSphere. 2025 Apr 16;10(5):e00940-24. doi: 10.1128/msphere.00940-24 (PMC12108064; doi:10.1128/msphere.00940-24)
Supplement: Supplemental figures, part 1 — Fig. S1 to S6. [file msphere.00940-24-s0001.pdf]

<u>Figure S1.</u> Results of the two independent 4CMenB vaccination and Ngo vaginal challenge experiments performed in female BALB/c mice. The first (**a**) and second (**b**) vaccination and challenge study. (top) Kaplan-Meier curves showing the percentage of animals colonized in each group (4CMenB, blue line; alum control, black) on each day post challenge. (Middle, left) The total number of days colonized for each animal. The Box plot denotes the median and upper/lower quartiles within the grey area with the whiskers extending to the minimum and maximum values. (Middle, right) The cumulative bacterial load recovered from each animal throughout the experiment. (Bottom) The vaginal bacterial load recovered from each animal on each day. Each symbol represents one animal. The alum control group is shown in black. The 4CMenB-vaccinated group is separated into green circles (4CMenB-vaccinated protected; no bacterial recovered from vaginal lavages on at minimum days four through six) or yellow circles (not protected 4CMenB-vaccinated animals; bacteria recovered from vaginal lavage on one or more of the last three days of the experiment) (middle, bottom). Red horizontal lines denote the median and symbols within the grey area denote no Ngo recovery (middle and bottom).

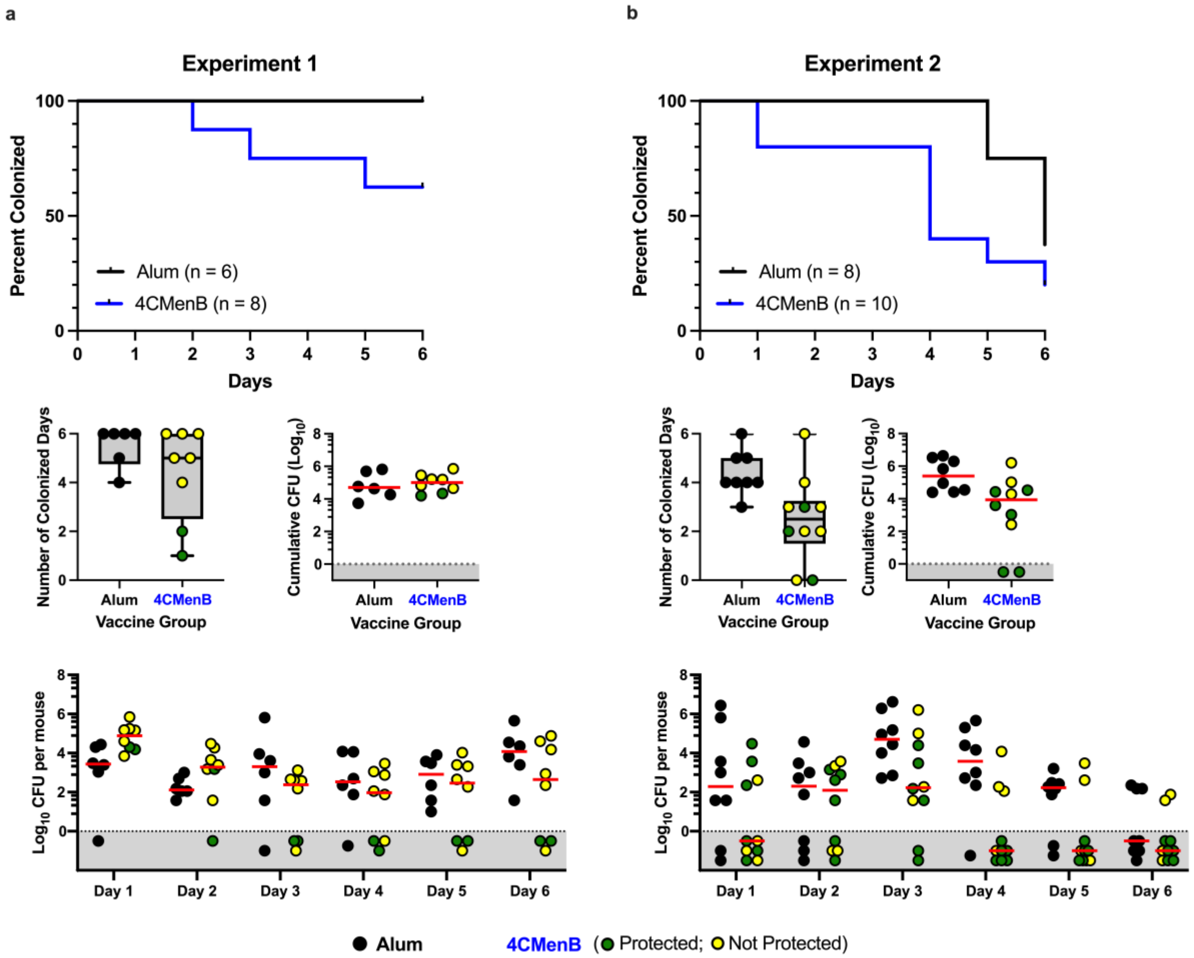

a
Experiment 1
Percent Colonized
Alum (n = 6)
4CMenB (n = 8)
Days
Number of Colonized Days
Cumulative CFU (Log10)
Alum
4CMenB
Vaccine Group
Log10 CFU per mouse
Day 1
Day 2
Day 3
Day 4
Day 5
Day 6
b
Experiment 2
Alum (n = 8)
4CMenB (n = 10)
Alum
4CMenB (Protected; Not Protected)

Figure S2. **Comparison of colonization rates and cumulative CFU between alum controls with protected and not protected 4CMenB-vaccinated animals**. 4CMenB vaccinated animals were separated into protected (green) and not protected (yellow) based off whether any amount of Ngo was recovered from vaginal lavages after day three of challenge. Evaluation of number of colonized days (**a**) and cumulative CFU (**b**). Non-parametric Kruskal-Wallis test was performed to compare groups in both data sets and p values are listed with red horizontal times indicating the media of each group. (**c**) Simple linear regression of number of colonized days against cumulative CFU with the R squared value listed and the p value from the F test indicating the slope of the line of best fit is significantly non-zero. Alum control animals are in black, and each symbol represents one animal.

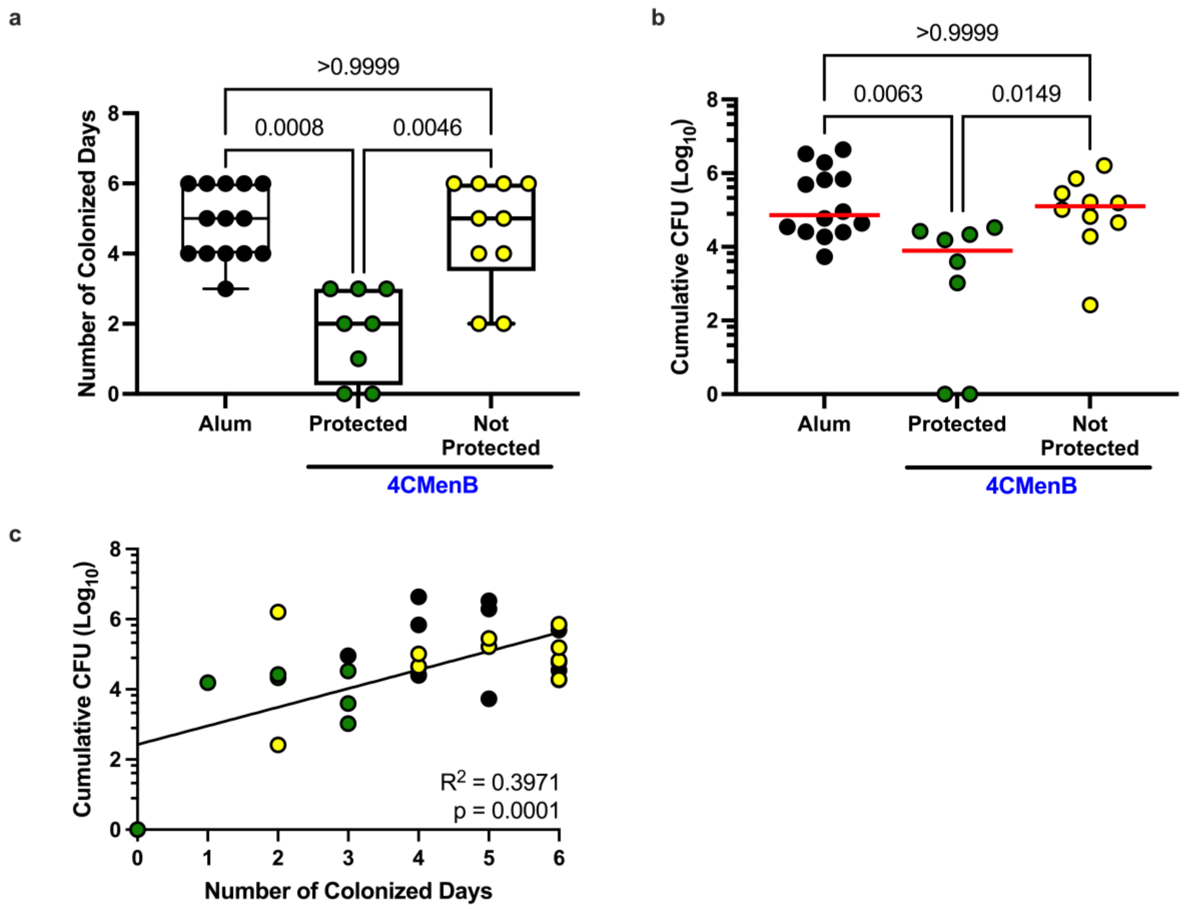
a
>0.9999
0.0008
0.0046
Number of Colonized Days
8
6
4
2
0
Alum
Protected
Not Protected
4CMenB
b
>0.9999
0.0063
0.0149
Cumulative CFU (Log_{10})
8
6
4
2
0
Alum
Protected
Not Protected
4CMenB
c
Cumulative CFU (Log_{10})
8
6
4
2
0
R^2 = 0.3971
p = 0.0001
0
1
2
3
4
5
6
Number of Colonized Days

Figure S3. **4CMenB immunization generates antibodies that bind *Neisseria* species.** After three vaccinations, IgG antibodies from serum (**a, b**) or IgA from vaginal lavages (**c, d**) against heat killed Nme NZ98/254 (the strain used to generate the outer membrane vesicle portion of 4CMenB; **a, c**) or Ngo FA1090 (challenge strain from this study; **b, d**) were quantified. Mann-Whitney non-parametric tests were performed between alum (black) and 4CMenB (blue) or protected (green) and not protected (yellow) groups and significant p values are displayed. Horizontal red bars indicate the median value of each group, and each symbol indicates one animal from the study.

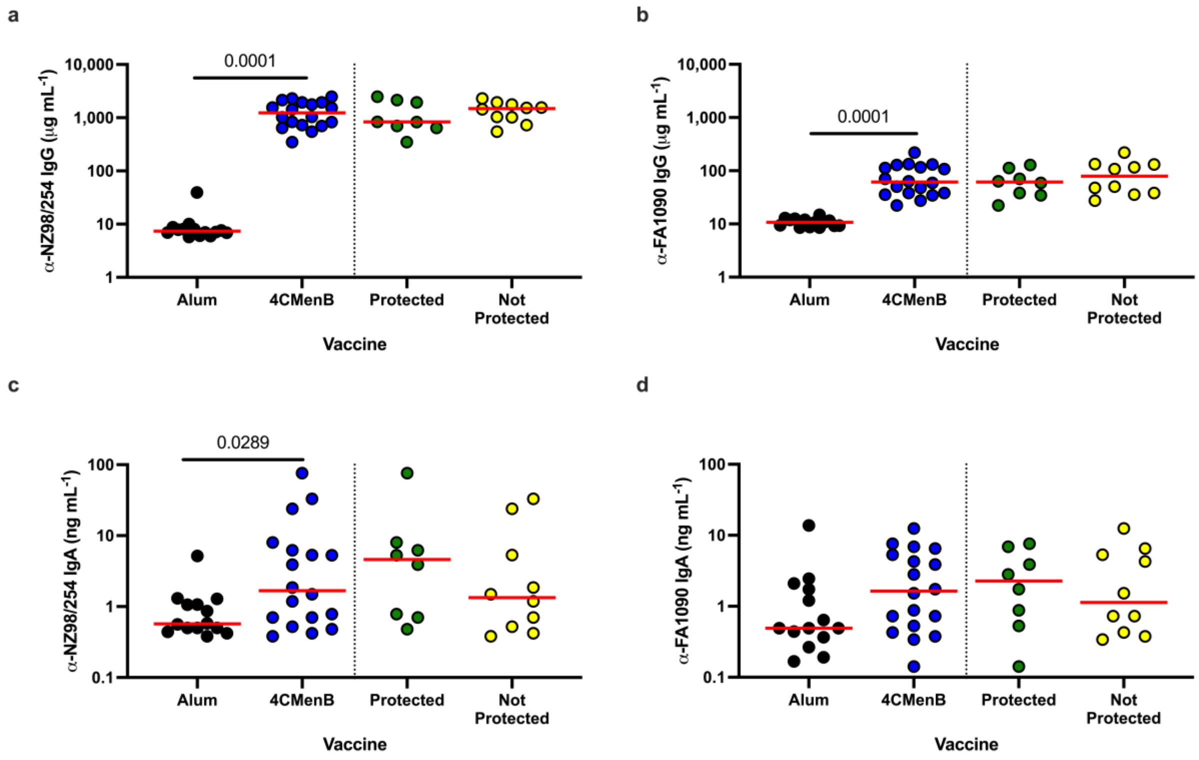

a
0.0001
α-NZ98/254 IgG (μg mL-1)
10,000
1,000
100
10
1
Alum
4CMenB
Protected
Not Protected
Vaccine
b
0.0001
α-FA1090 IgG (μg mL-1)
10,000
1,000
100
10
1
Alum
4CMenB
Protected
Not Protected
Vaccine
c
0.0289
α-NZ98/254 IgA (ng mL-1)
100
10
1
0.1
Alum
4CMenB
Protected
Not Protected
Vaccine
d
α-FA1090 IgA (ng mL-1)
100
10
1
0.1
Alum
4CMenB
Protected
Not Protected
Vaccine

Figure S4. Anti-Neisserial antibodies do not correlate with protection. Simple linear regression analyses were performed between serum IgG antibodies (**a, b, e, f**) or vaginal IgA antibodies (**c, d, g, h**) against heat killed Nme NZ98/254 (the strain used to generate the outer membrane vesicle portion of 4CMenB; **a, c, e, g**) or *Ngo* FA1090 (challenge strain from this study; **b, d, f, h**) and cumulative bacterial CFU (**a – d**) or the number of CFU positive days (**e – h**). Each symbol represents one animal, and each group is indicated (alum, black; 4CMenB protected, green; 4CMenB not protected, yellow). The line of best fit is present on each graph and the R squared value is listed. Statistical F tests were performed on each graph; however, no slope was significantly non-zero.

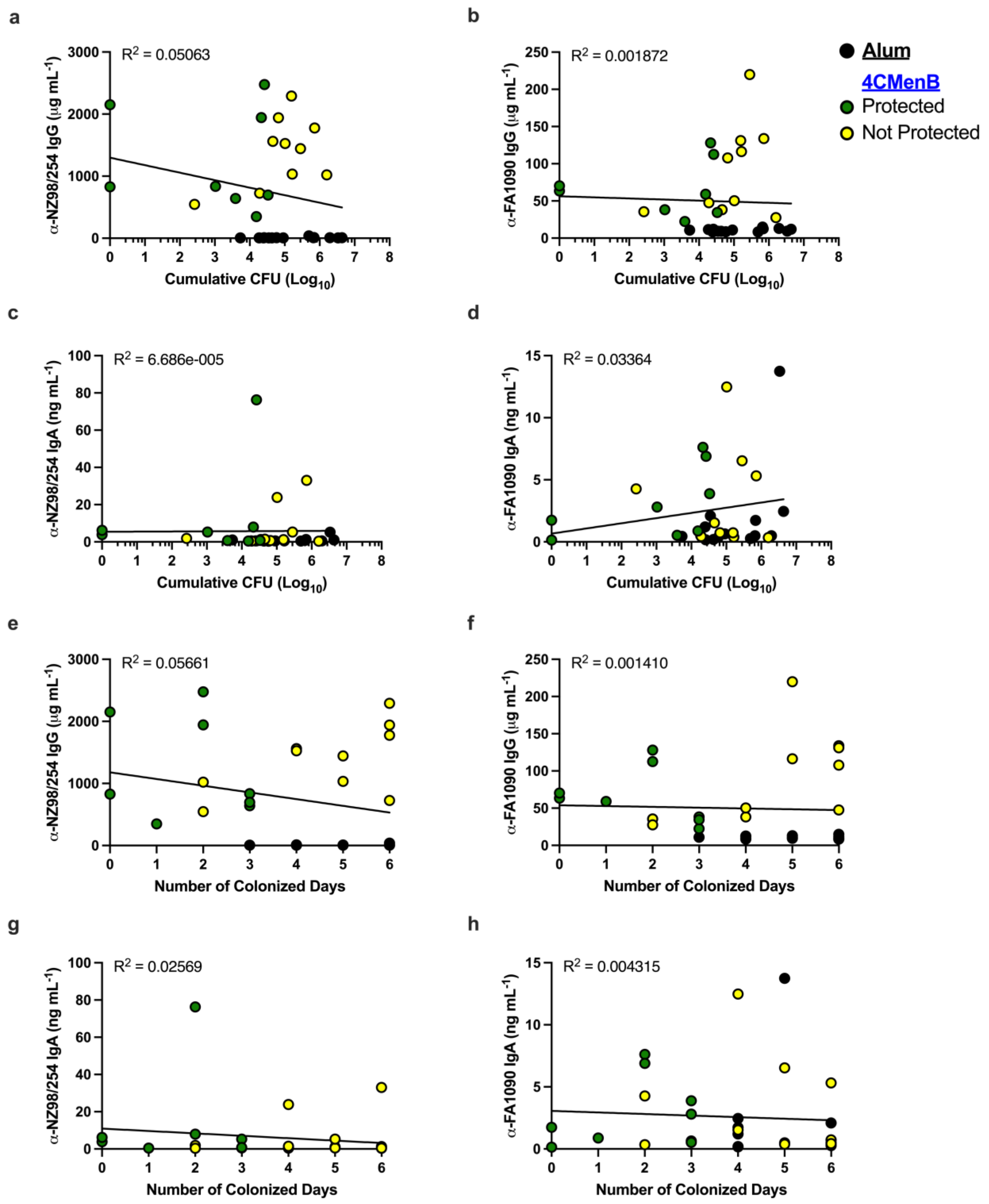

a
R² = 0.05063
α-NZ98/254 IgG (μg mL⁻¹)
Cumulative CFU (Log10)
b
R² = 0.001872
α-FA1090 IgG (μg mL⁻¹)
Cumulative CFU (Log10)
Alum
4CMenB
Protected
Not Protected
c
R² = 6.686e-005
α-NZ98/254 IgA (ng mL⁻¹)
Cumulative CFU (Log10)
d
R² = 0.03364
α-FA1090 IgA (ng mL⁻¹)
Cumulative CFU (Log10)
e
R² = 0.05661
α-NZ98/254 IgG (μg mL⁻¹)
Number of Colonized Days
f
R² = 0.001410
α-FA1090 IgG (μg mL⁻¹)
Number of Colonized Days
g
R² = 0.02569
α-NZ98/254 IgA (ng mL⁻¹)
Number of Colonized Days
h
R² = 0.004315
α-FA1090 IgA (ng mL⁻¹)
Number of Colonized Days

Figure S5. Distinct IgG subclasses react against *Neisseria* species following 4CMenB-immunization. IgG1, IgG2a, IgG2b, IgG3 subclasses in terminal serum of 4CMenB- and alum-immunized mice against heat killed Nme NZ98/254 (**a - d**) or Ngo FA1090 (**e - h**) were measured. Mann-Whitney non-parametric tests were performed between alum (black) and Bexsero (blue) or protected (green) and not protected (yellow) groups and significant p values are displayed (left panel). Horizontal red bars indicate the median value of each group, and each symbol indicates one animal from the study. Simple linear regression analyses were performed between IgG1 (**a, e**), IgG2a (**b, f**) IgG2b (**c, g**) and IgG3 (**d, h**) and the number of CFU positive days (middle panel) or cumulative bacterial CFU (right panel) is shown. Each symbol represents one animal. The line of best fit is present on each graph and the R squared value is listed. Statistical F tests were performed on each graph; and p-values for significantly non-zero slopes depicted.

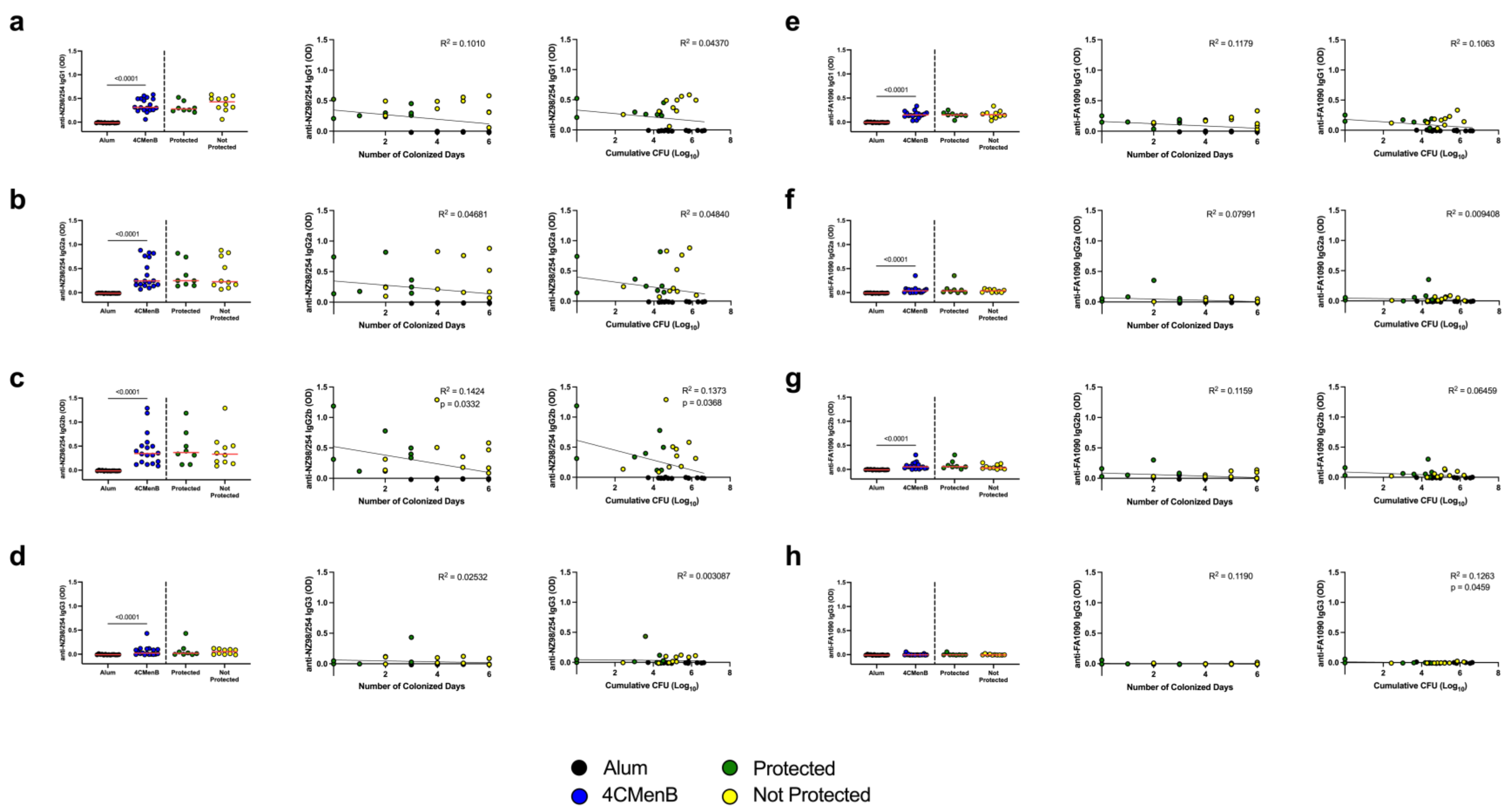

a
b
c
d
e
f
g
h
anti-NZ98/254 IgG1 (OD)
anti-NZ98/254 IgG2a (OD)
anti-NZ98/254 IgG2b (OD)
anti-NZ98/254 IgG3 (OD)
anti-FA1090 IgG1 (OD)
anti-FA1090 IgG2a (OD)
anti-FA1090 IgG2b (OD)
anti-FA1090 IgG3 (OD)
Alum
4CMenB
Protected
Not Protected
<0.0001
Number of Colonized Days
Cumulative CFU (Log10)
R2 = 0.1010
R2 = 0.04370
R2 = 0.04681
R2 = 0.04840
R2 = 0.1424
p = 0.0332
R2 = 0.1373
p = 0.0368
R2 = 0.02532
R2 = 0.003087
R2 = 0.1179
R2 = 0.1063
R2 = 0.07991
R2 = 0.009408
R2 = 0.1159
R2 = 0.06459
R2 = 0.1190
R2 = 0.1263
p = 0.0459

Figure S6. Variability in band recognition pattern among 4CMenB-immunized animals and potential correlations. Western blots using terminal serum from individual 4CMenB-immunized mice with the identifier indicated above, and pooled sera from alum-immunized mice, against whole bacterial lysates. For each blot, lane 1 – molecular weight marker (245kDa, 180kDa, 135kDa, 100kDa, 75kDa-red, 63kDa, 48kDa, 35kDa, 25kDa-green, 20kDa, top to bottom); lane 2 - Nme NZ98/254; lane 3 – Ngo FA1090 (**a, b** – zoomed to depict individual FA1090 bands quantified by densitometry). Pearson correlation coefficient was calculated to identify potential linear relationships between intensity for each band at 2 min and 8 min exposures and number of colonized days and cumulative CFU. Pearson r values depicted as a heatmap (c); colour scale represents Pearson r values (red – positive relationship; blue – negative relationship). Interactions that could not be computed are grey. p values < 0.05 indicated with *; p values <0.1 also noted. Linear regression analyses of band intensity versus number of days colonized depicted for significant interactions only, along with comparison of intensities between protected and not protected using non-parametric Mann-Whitney test (**d** – Band 3, **e** – Band 6). For scatter plots, the line regression line, R^2 (goodness of fit) and p values depicted.

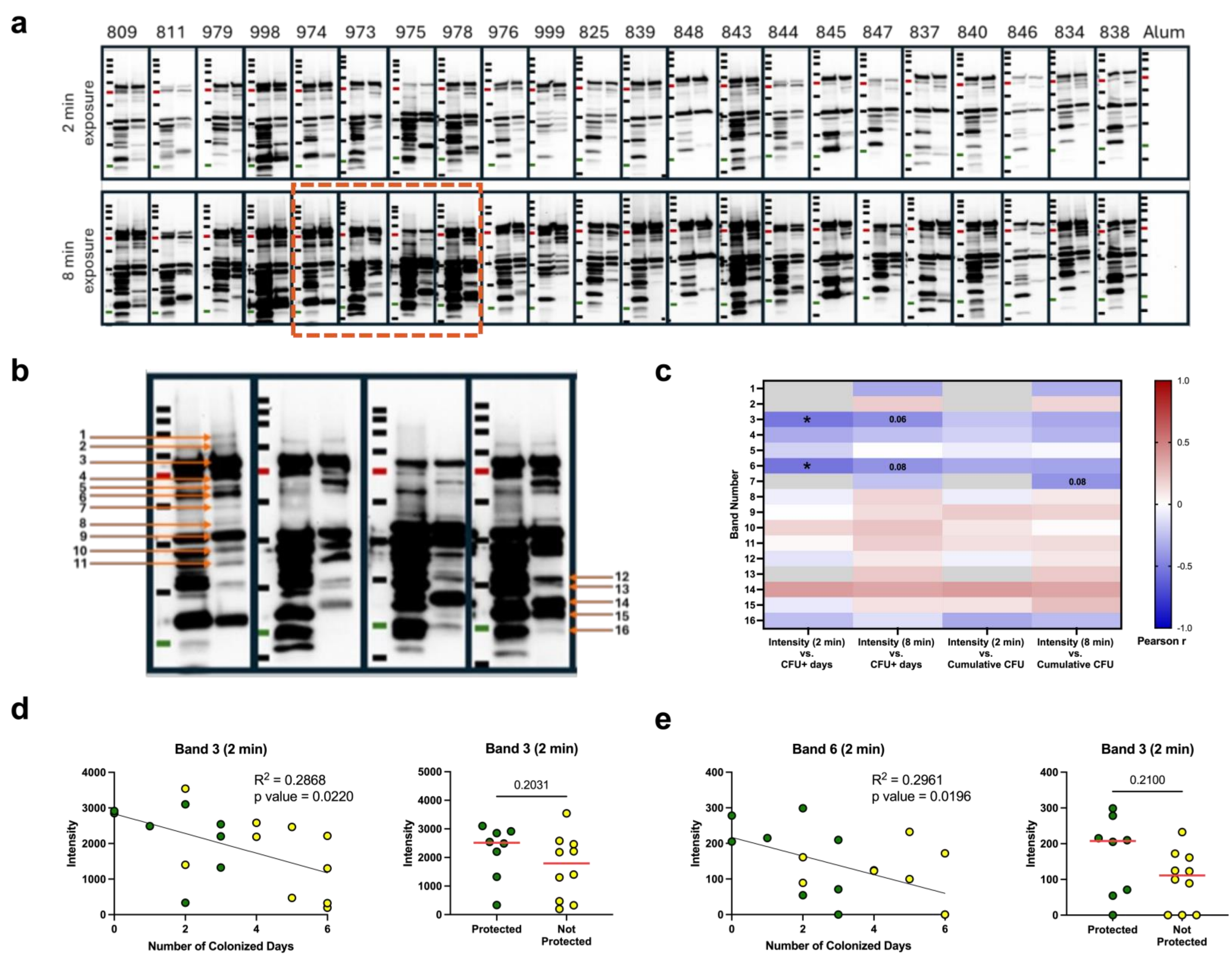

a
809 811 979 998 974 973 975 978 976 999 825 839 848 843 844 845 847 837 840 846 834 838 Alum
2 min exposure
8 min exposure
b
1 2 3 4 5 6 7 8 9 10 11
12 13 14 15 16
c
Band Number
Intensity (2 min) vs. CFU+ days
Intensity (8 min) vs. CFU+ days
Intensity (2 min) vs. Cumulative CFU
Intensity (8 min) vs. Cumulative CFU
0.06
0.08
0.08
Pearson r
1.0 0.5 0 -0.5 -1.0
d
Band 3 (2 min)
R^2 = 0.2868
p value = 0.0220
Intensity
Number of Colonized Days
Band 3 (2 min)
0.2031
Protected
Not Protected
e
Band 6 (2 min)
R^2 = 0.2961
p value = 0.0196
Number of Colonized Days
Band 3 (2 min)
0.2100
Protected
Not Protected
