# Supplementary material for: Meningococcal vaccine 4CMenB elicits a robust cellular immune response that targets but is not consistently protective against Neisseria gonorrhoeae during murine vaginal infection
Source: mSphere. 2025 Apr 16;10(5):e00940-24. doi: 10.1128/msphere.00940-24 (PMC12108064; doi:10.1128/msphere.00940-24)
Supplement: Supplemental figures, part 2 — Fig. S7 to S12. [file msphere.00940-24-s0002.pdf]

__Figure S7.__ **4CMenB-immunization did not augment serum bactericidal activity (SBA) titres against FA1090.** Log-phase Ngo FA1090 was incubated with increasing 2-fold dilutions of heat inactivated 4CMenB or alum-immunized terminal serum and 2.5% baby rabbit complement (**a, b**) or 15% pooled human serum (**c, d**). CFU recovered after 60 minutes of incubation (**a, c**) and SBA titre, which is the highest dilution at which 50% killing was observed relative to no antibody control, (**b, d**) are graphed. Each symbol depicts serum from an individual animal; alum (black) and Bexsero (blue); Bexsero protected (green) and not protected (yellow); line at median. Dotted line (**a ,c**) represents the limit for obtaining individual CFU counts; dotted lines (**b, d**) represent the highest and lowest mouse serum dilutions tested. Two-way ANOVA with Šídák's multiple comparisons test to detect differences in CFU recovered in alum versus 4CMenB groups for each serum dilution (for **a**) and non-parametric Mann-Whitney test comparing alum versus 4CMenB or 4CMenB Protected versus Not Protected groups (for **b**) did not yield significant p values.

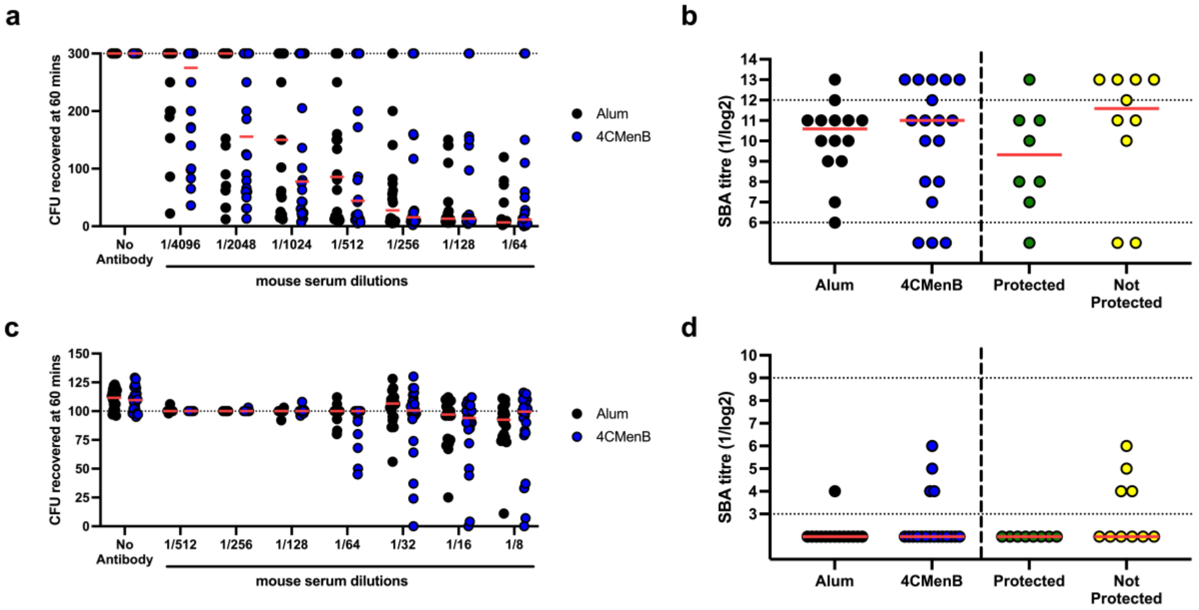

a
CFU recovered at 60 mins
300
200
100
0
No Antibody
1/4096
1/2048
1/1024
1/512
1/256
1/128
1/64
mouse serum dilutions
Alum
4CMenB
b
SBA titre (1/log2)
14
13
12
11
10
9
8
7
6
Alum
4CMenB
Protected
Not Protected
c
CFU recovered at 60 mins
150
125
100
75
50
25
0
No Antibody
1/512
1/256
1/128
1/64
1/32
1/16
1/8
mouse serum dilutions
Alum
4CMenB
d
SBA titre (1/log2)
10
9
8
7
6
5
4
3
Alum
4CMenB
Protected
Not Protected

Figure S8. Flow cytometry gating strategy. Single cell suspensions of lymphocytes from the spleen or the genital tract from vaccinated and infected animals were cultured with either media alone or 4CMenB in the presence of a Golgi apparatus-blocking chemical to perform surface and intracellular flow cytometry. Cells were first gated upon as single, live (viability dye negative), leukocytes (CD45+). They were then gated on by forward and side scatter for the correct lymphocyte morphology. T cells (CD3+), B cells (CD19+) and non-T non-B lymphocytes (CD3- CD19-) were all sub-gated upon for further analysis. T cells were separated into CD4+ and CD8α+ followed by either memory markers (naïve, CD44- CD62L+; central memory [Tcm], CD44+ CD62L+; effector memory [Tem], CD44+ CD62L-) or for cytokine positivity (CD69+ plus any one of seven cytokine markers). Non-T non-B lymphocytes were gated on for cytokine positivity. Only surface gating was performed on genital tract lymphocytes.

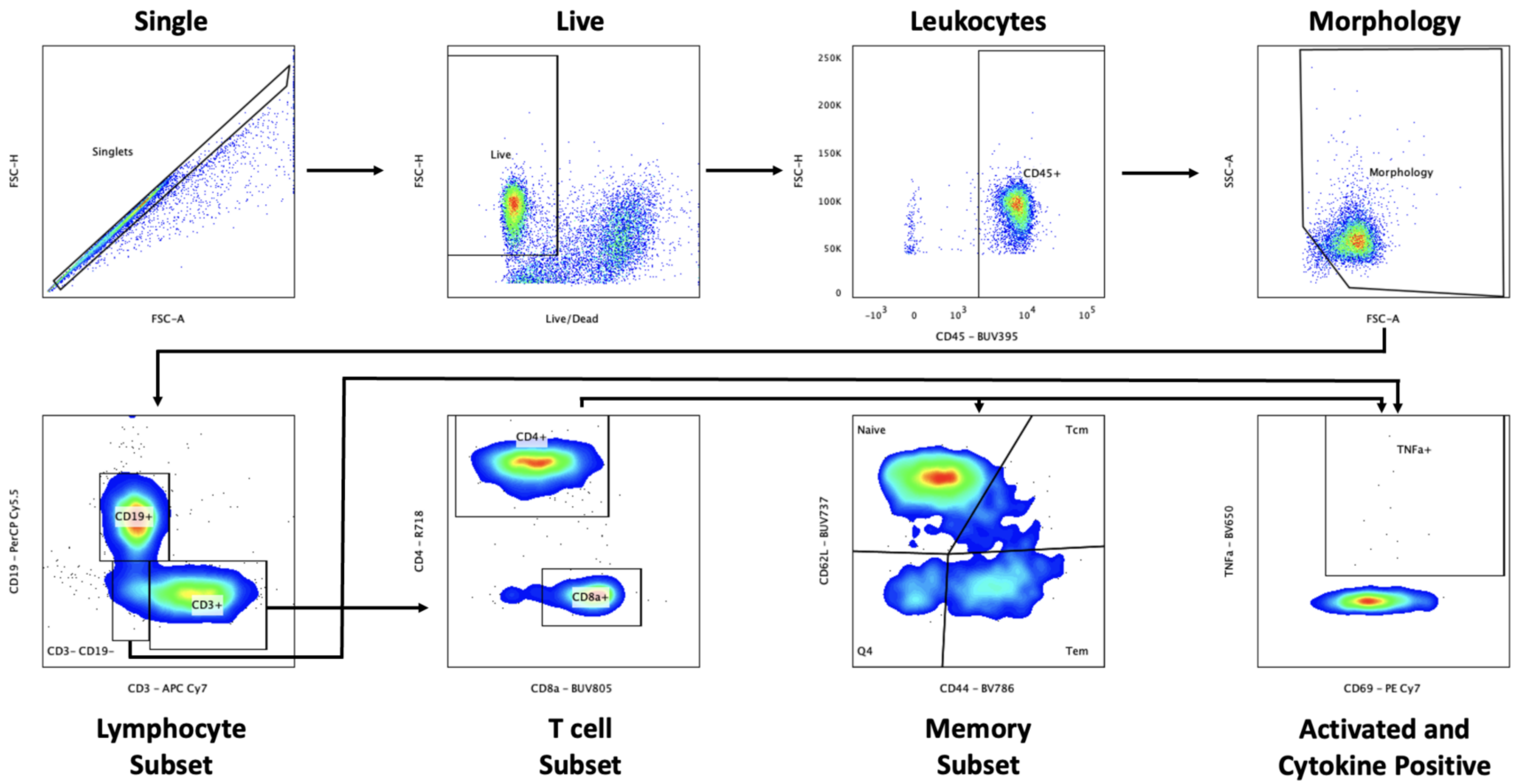
Single
Singlets
FSC-H
FSC-A
Live
Live
FSC-H
Live/Dead
Leukocytes
250K
200K
150K
100K
50K
0
CD45+
FSC-H
-10^3
0
10^3
10^4
10^5
CD45 - BUV395
Morphology
Morphology
SSC-A
FSC-A
CD19+
CD3+
CD3- CD19-
CD19 - PerCP Cy5.5
CD3 - APC Cy7
Lymphocyte Subset
CD4+
CD8a+
CD4 - R718
CD8a - BUV805
T cell Subset
Naive
Tcm
Q4
Tem
CD62L - BUV737
CD44 - BV786
Memory Subset
TNFa+
TNFa - BV650
CD69 - PE Cy7
Activated and Cytokine Positive

Figure S9. An increase in the total number of CD3- CD19- T_H1+ cells per gram is observed in 4CMenB vaccinated mouse spleens. Splenocytes from necropsied animals on day six post-challenge were stimulated to assess the vaccine-specific immune response which was measured using intracellular cytokine staining and flow cytometry. Each column is the measurement of the total number of cells per gram secreting a specific cytokine (or combination of any of the T_H1 cytokines [IFNγ, IL-2 or TNFα] as determined by Boolean gating) after cells were stimulated with either media alone (black) or with 4CMenB (white). Rows indicate both the vaccine group being assessed (black, alum; blue, 4CMenB) as well as the cell subset (CD4+, CD8+ or CD3- CD19- lymphocytes). Each symbol is one animal, and the red horizontal bar represents the median of each data set. Non-parametric (Mann-Whitney) analysis was performed both within the vaccine group (between media and 4CMenB stimulation) and between groups (the alum- or 4CMenB-stimulated cells compared between alum and 4CMenB vaccinated animals). Significant p values are listed with a horizonal bar for within group comparison or in the of the graph for between vaccine groups.

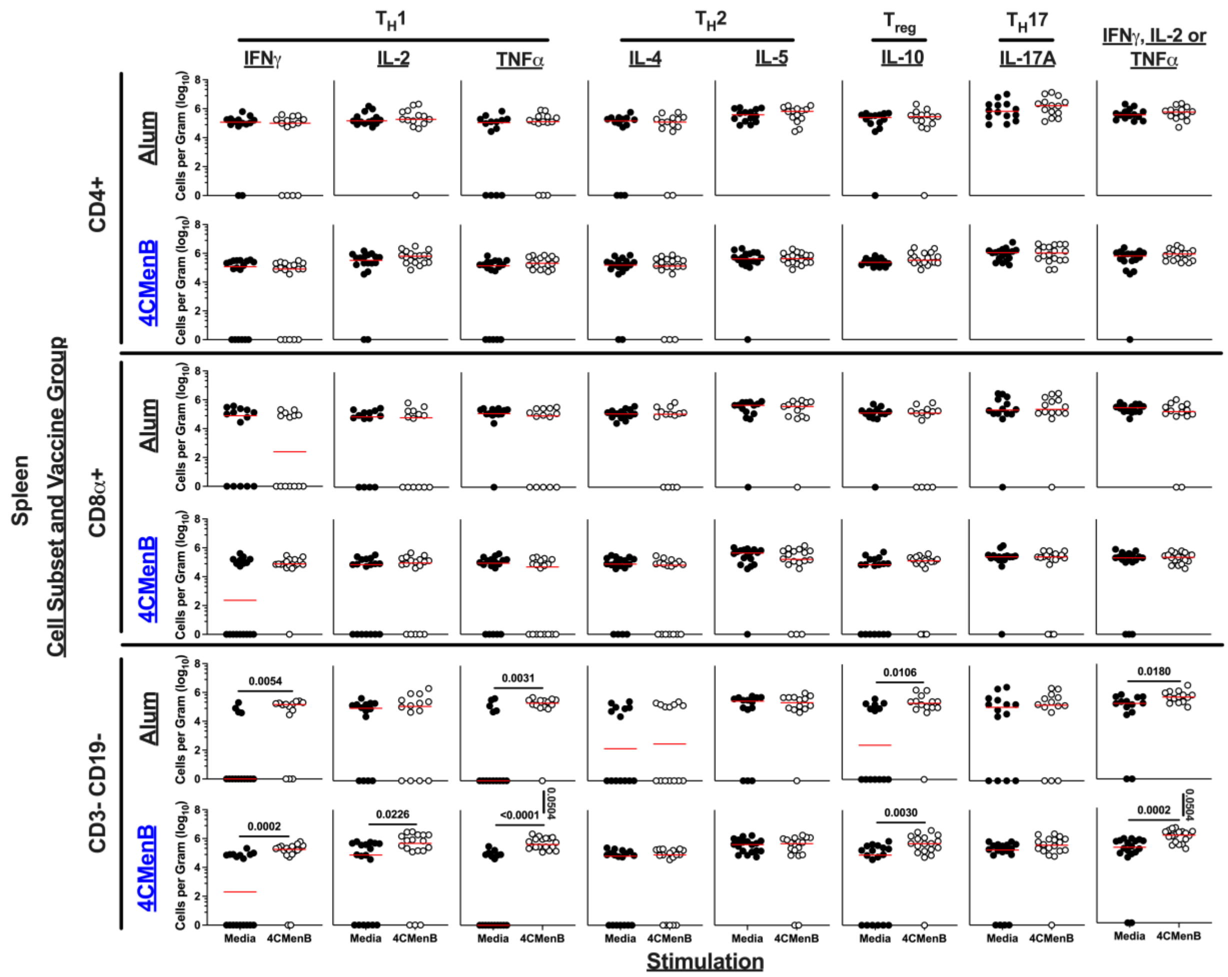

TH1
TH2
Treg
TH17
IFNγ
IL-2
TNFα
IL-4
IL-5
IL-10
IL-17A
IFNγ, IL-2 or TNFα
Spleen
Cell Subset and Vaccine Group
CD4+
CD8α+
CD3- CD19-
Alum
4CMenB
Cells per Gram (log10)
0.0054
0.0031
0.0106
0.0180
0.0002
0.0226
<0.0001
0.0504
0.0030
0.0002
0.0504
Media
4CMenB
Stimulation

Figure S10. **Percent of parent population T cell immune response in protected and unprotected splenocytes from 4CMenB vaccinated mice.** Splenocytes from necropsied animals on day six post-challenge were stimulated to assess the vaccine-specific immune response which was measured using intracellular cytokine staining and flow cytometry. Each column is the frequency measurement of a specific cytokine (or a combination of any of the T_H1 cytokines [IFNγ, IL-2 or TNFα as performed by Boolean gating] in the far-right column) after cells were stimulated with either media alone (black) or with 4CMenB (white). Rows indicate both which protection group was being assessed (yellow, not protected 4CMenB vaccinated; green, protected 4CMenB vaccinated) and which cell subset (CD4+, CD8+, or CD3- CD19- lymphocytes). Each symbol is an animal, and the red horizontal bar represents the median of each data set. Non-parametric (Mann-Whitney) analysis was performed both within the vaccine groups (between media and 4CMenB stimulation) and between groups (the alum or 4CMenB stimulated cells compared between alum and 4CMenB vaccinated animals). Significant p values are listed with a horizonal black bar if it was within groups or a vertical black bar if it was between vaccine groups.

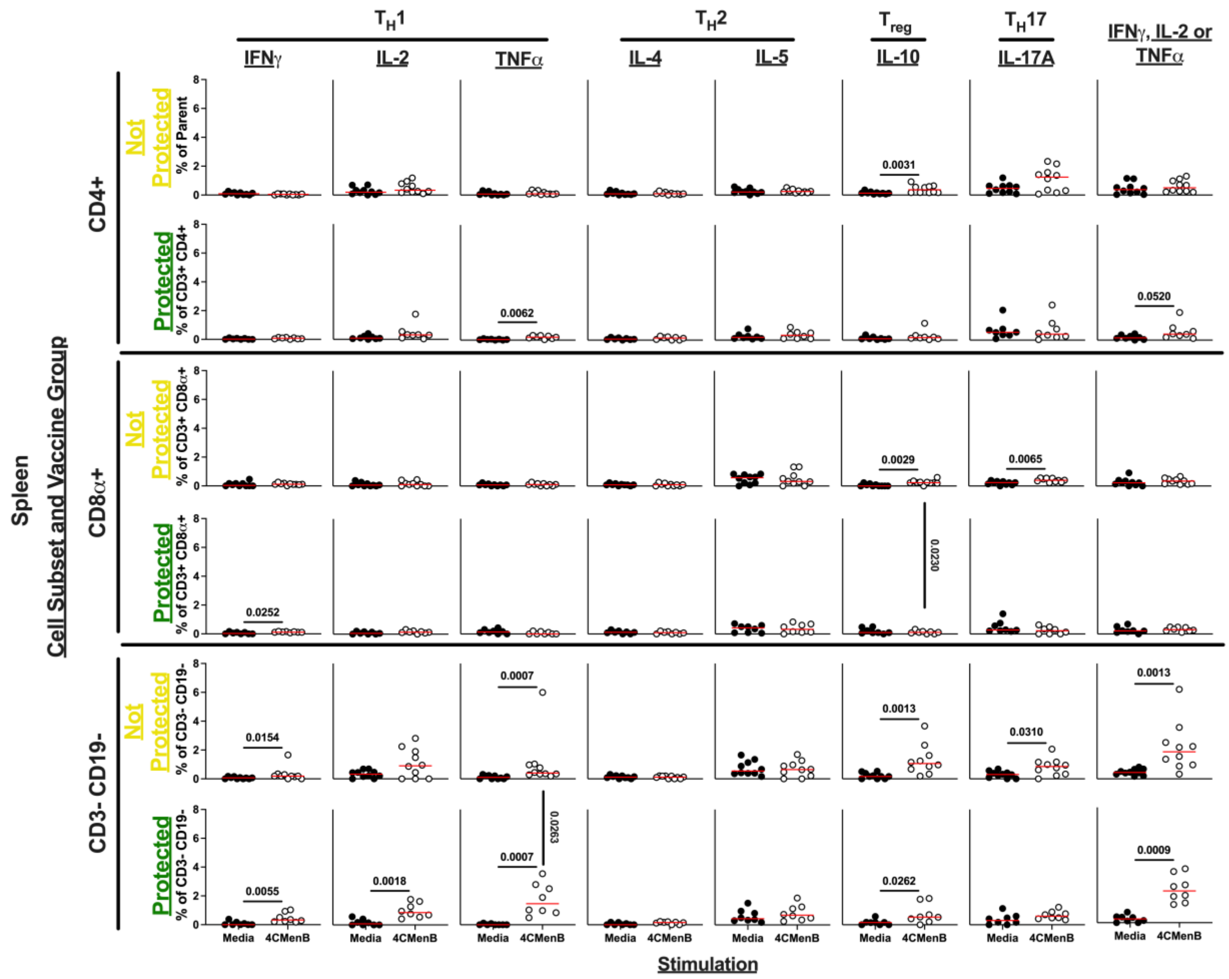

TH1
TH2
Treg
TH17
IFNγ
IL-2
TNFα
IL-4
IL-5
IL-10
IL-17A
IFNγ, IL-2 or TNFα
Spleen
Cell Subset and Vaccine Group
CD4+
CD8α+
CD3- CD19-
Not Protected
Protected
% of Parent
% of CD3+ CD4+
% of CD3+ CD8α+
% of CD3- CD19-
0.0031
0.0062
0.0520
0.0029
0.0065
0.0252
0.0230
0.0154
0.0007
0.0013
0.0310
0.0013
0.0055
0.0018
0.0263
0.0007
0.0262
0.0009
Media
4CMenB
Stimulation

<u>Figure S11.</u> Cells per gram of tissue T cell immune response in protected and unprotected splenocytes from 4CMenB vaccinated mice. Splenocytes from necropsied animals on day six post-challenge were stimulated to assess the vaccine-specific immune response which was measured using intracellular cytokine staining and flow cytometry. Each column is the measurement of the total number of cells per gram secreting a specific cytokine (or combination of any of the T_H1 cytokines [IFNγ, IL-2 or TNFα] as determined by Boolean gating) after cells were stimulated with either media alone (black) or with 4CMenB (white). Rows indicate both the vaccine group being assessed (yellow, not protected 4CMenB vaccinated animals; green, protected 4CMenB vaccinated animals) as well as the cell subset (CD4+, CD8+ or CD3- CD19- lymphocytes). Each symbol is one animal, and the red horizontal bar represents the median of each data set. Non-parametric (Mann-Whitney) analysis was performed both within the vaccine group (between media and 4CMenB stimulation) and between groups (the alum- or 4CMenB-stimulated cells compared between alum and 4CMenB vaccinated animals). Significant p values are listed with a horizonal bar for within group comparison or in the of the graph for between vaccine groups.

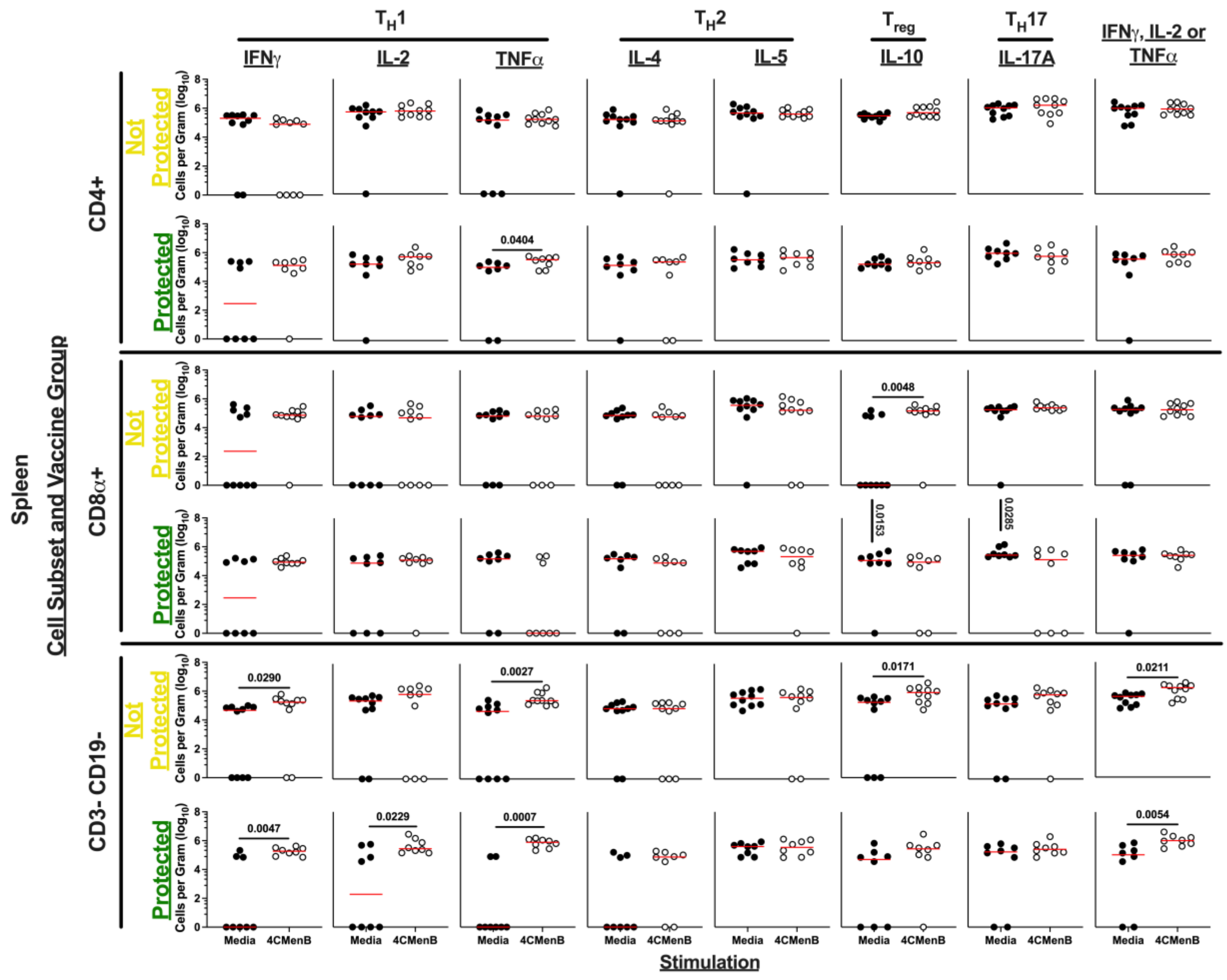

TH1
TH2
Treg
TH17
IFNγ
IL-2
TNFα
IL-4
IL-5
IL-10
IL-17A
IFNγ, IL-2 or TNFα
Spleen
Cell Subset and Vaccine Group
CD4+
CD8α+
CD3- CD19-
Not Protected
Protected
Cells per Gram (log10)
0.0404
0.0048
0.0153
0.0285
0.0290
0.0027
0.0171
0.0211
0.0047
0.0229
0.0007
0.0054
Media
4CMenB
Stimulation

Figure S12. Boolean gating on CD3- CD19- splenocytes producing TH1 cytokines. Splenocytes from control and 4CMenB-vaccinated animals were stimulated with media alone or Bexsero and analyzed by Boolean gating flow cytometry to assess non-T non-B (CD3- CD19-) lymphocytes that were expressing any combination of T_H1 cytokines (IFNγ, IL-2 and/or TNFα). (**a**) Comparison of alum (black) versus all 4CMenB-vaccinated (blue) animals by percent (left) or cells per gram of tissue (right). (**b**) Comparison of protected (green) versus not protected (yellow) 4CMenB-vaccinated animals by percent (left) or cells per gram of tissue (right). Non-parametric (Mann-Whitney) analysis was performed both within the groups (between media and 4CMenB stimulation) and between groups (the alum- or 4CMenB-stimulated cells compared between alum and 4CMenB vaccinated animals or protected and not protected 4CMenB-vaccinated animals). Horizonal red bars indicate the median of each data set and horizontal black bars with numbers above indicate the groups being statistically compared and the statistically significant p value as determined by Mann-Whitney analysis.

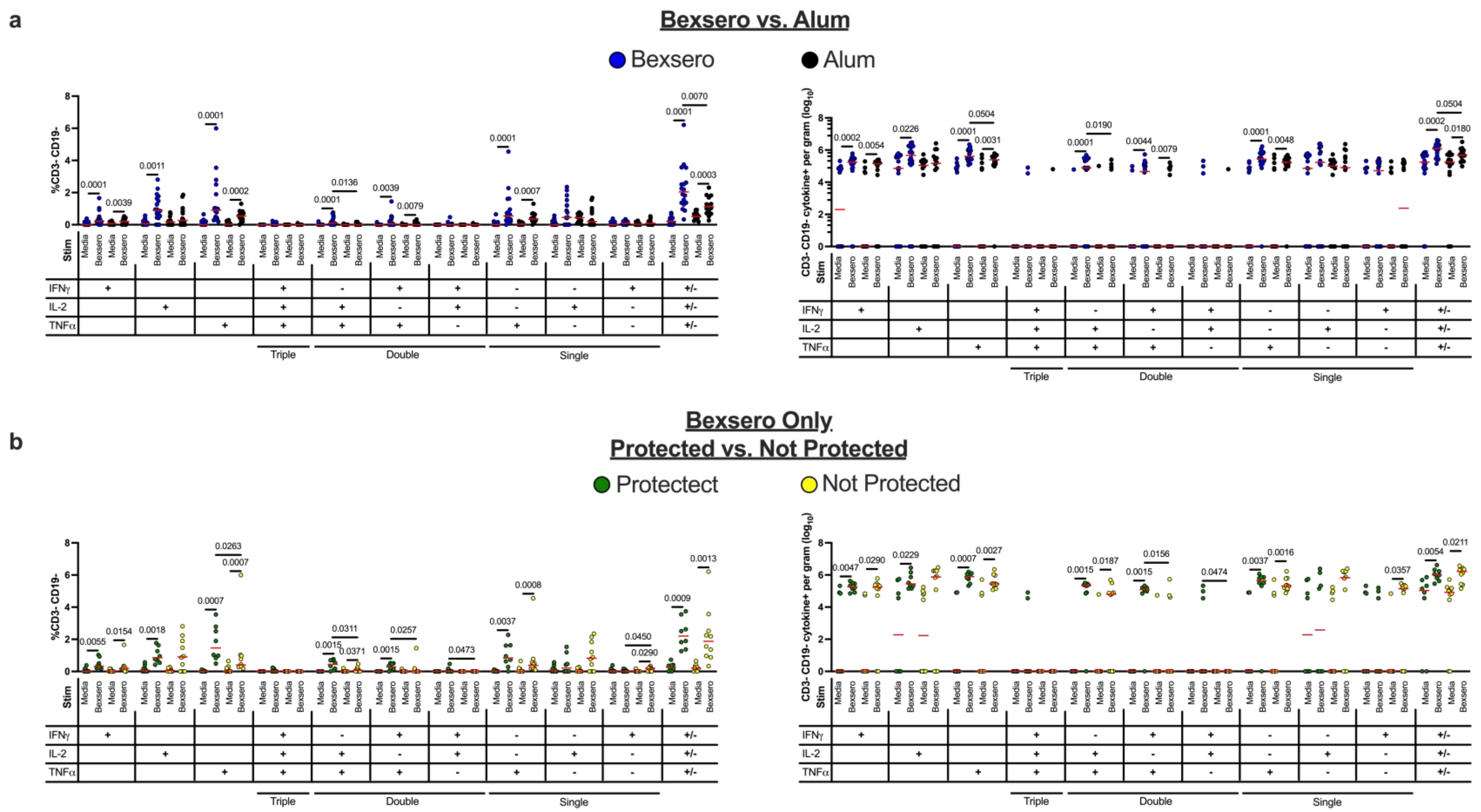
a
Bexsero vs. Alum
Bexsero
Alum
%CD3- CD19-
CD3- CD19- cytokine+ per gram (log10)
Stim
IFNγ
IL-2
TNFα
Triple
Double
Single
b
Bexsero Only
Protected vs. Not Protected
Protectect
Not Protected
