# Supplementary material for: Meningococcal vaccine 4CMenB elicits a robust cellular immune response that targets but is not consistently protective against Neisseria gonorrhoeae during murine vaginal infection
Source: mSphere. 2025 Apr 16;10(5):e00940-24. doi: 10.1128/msphere.00940-24 (PMC12108064; doi:10.1128/msphere.00940-24)

<u>Figure S13.</u> Lymphocytes from the genital tract of 4CMenB vaccinated animals secrete more IL-2, IL-4 and IL-17A upon stimulation than their alum vaccinated counterparts. Lymphocytes from the genital tract of vaccinated animals were incubated with either media alone or 4CMenB and the secreted cytokine response from the culture media were measured using a T cell centric 18-plex. Top, a heatmap of normalized values for each cytokine listed from when cells were stimulated with the indicated agent (columns) from the specific group (top row, alum vaccinated animals; second row, all 4CMenB vaccinated animals; third row, protected 4CMenB animals; bottom row, not protected 4CMenB vaccinated animals). Bottom, Scatterplots of the raw cytokine data used to generate the heat map. Each symbol represents the measure of an individual animal's cells after stimulation with either media (black) or 4CMenB (blue). Groups are listed on the X-axis. Red horizontal lines indicated the median values, and black horizontal lines connect two groups with statistically different medians (using Mann-Whitney non-parametric comparison) with p values listed above.

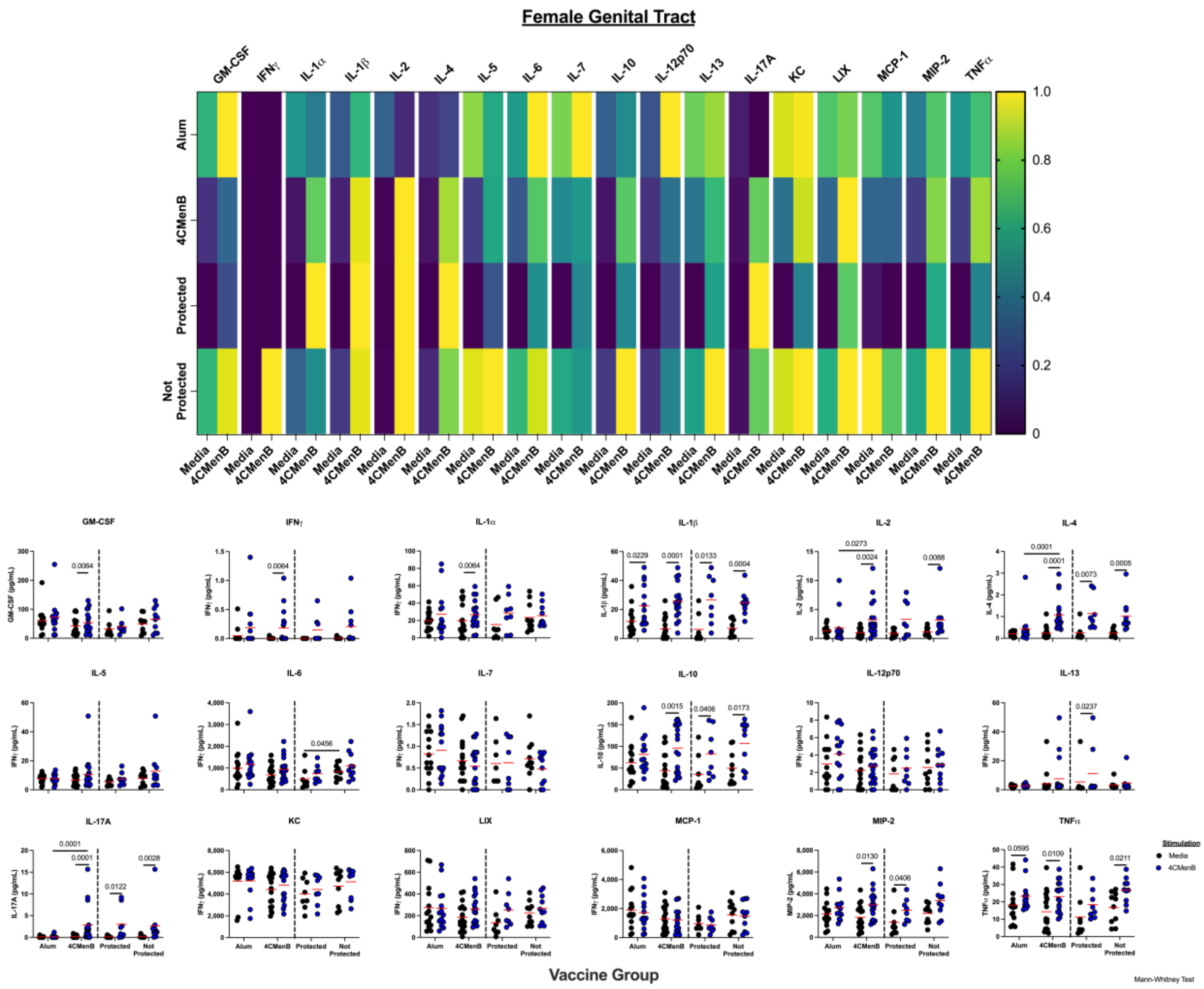
Female Genital Tract
GM-CSF
IFNγ
IL-1α
IL-1β
IL-2
IL-4
IL-5
IL-6
IL-7
IL-10
IL-12p70
IL-13
IL-17A
KC
LIX
MCP-1
MIP-2
TNFα
Alum
4CMenB
Protected
Not Protected
Media
4CMenB
1.0
0.8
0.6
0.4
0.2
0
Alum
4CMenB
Protected
Not Protected
Stimulation
Media
4CMenB
Vaccine Group
Mann-Whitney Test

Figure S14. Multiplex analysis of vaginal lavages from prior to and during gonococcal challenge. Lavages from the vaginal lumen of all animals approximately two weeks post-vaccination (prior to challenge) and days three- and six post-challenge were assayed for 45 different cytokines using a multiplex assay. Left, a heat map of the normalized median values for each cytokine (indicated at the left) on each day (indicated on the left) in each specific group (indicated on the top). Right, scatter plots of all cytokines that were statistically different between the 4CMenB and alum vaccinated groups or protected or not protected 4CMenB-vaccinated groups as determined by Mann-Whitney non-parametric comparison. Each symbol is one animal (black, alum; blue, 4CMenB; protected Bexsero, green; not protected Bexsero, yellow) and red horizontal bars indicate the median value.

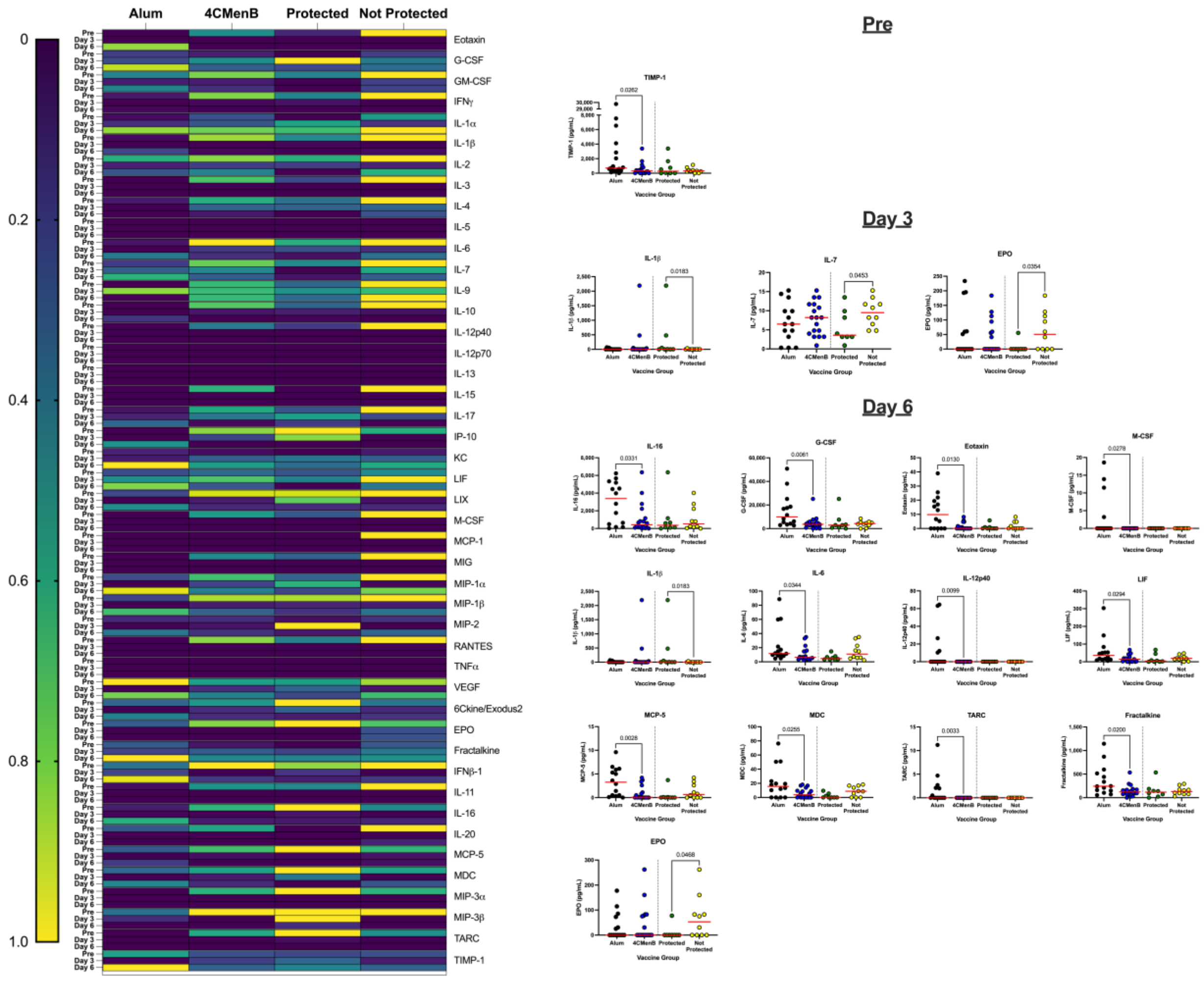
Alum
4CMenB
Protected
Not Protected
0
0.2
0.4
0.6
0.8
1.0
Pre
Day 3
Day 6
Eotaxin
G-CSF
GM-CSF
IFNγ
IL-1α
IL-1β
IL-2
IL-3
IL-4
IL-5
IL-6
IL-7
IL-9
IL-10
IL-12p40
IL-12p70
IL-13
IL-15
IL-17
IP-10
KC
LIF
LIX
M-CSF
MCP-1
MIG
MIP-1α
MIP-1β
MIP-2
RANTES
TNFα
VEGF
6Ckine/Exodus2
EPO
Fractalkine
IFNβ-1
IL-11
IL-16
IL-20
MCP-5
MDC
MIP-3α
MIP-3β
TARC
TIMP-1
Pre
TIMP-1
0.0262
Day 3
IL-1β
0.0183
IL-7
0.0453
EPO
0.0354
Day 6
IL-16
0.0331
G-CSF
0.0061
Eotaxin
0.0130
M-CSF
0.0278
IL-1β
0.0183
IL-6
0.0344
IL-12p40
0.0099
LIF
0.0294
MCP-5
0.0028
MDC
0.0255
TARC
0.0033
Fractalkine
0.0200
EPO
0.0468
Vaccine Group
Alum
4CMenB
Protected
Not Protected

<u>Figure S15.</u> T cell 18-plex Principal Component Analysis results in 4CMenB-stimulated spleen samples. (**a**) The first ten eigenvalues with individual percentages and cumulative percentages. The first three principal components (PC) explain nearly 81% of the variability of the data. (PC1 = 57.4%, PC2 = 14.3%, and PC3 = 9.0%). (**b**) Stack-plot of loading coefficients on the first 3 principal components. The x-axis shows cytokines and chemokines, the y-axis shows the magnitude and direction of the loading coefficients on PC1 (pink), PC2 (orange), and PC3 (yellow) for each variable. Note that individual loadings range between -1 (strongest possible negative correlation) and 1 (strongest possible positive correlation). Scores for PC1 (**c**), PC2 (**d**), and PC3 (**e**) comparing alum (black) and 4CMenB (blue) treated animal (left) and protected (green) and not protected (yellow) 4CMenB-vaccinated animals (right). Unpaired t-tests compared the groups with p-values < 0.05 shown.

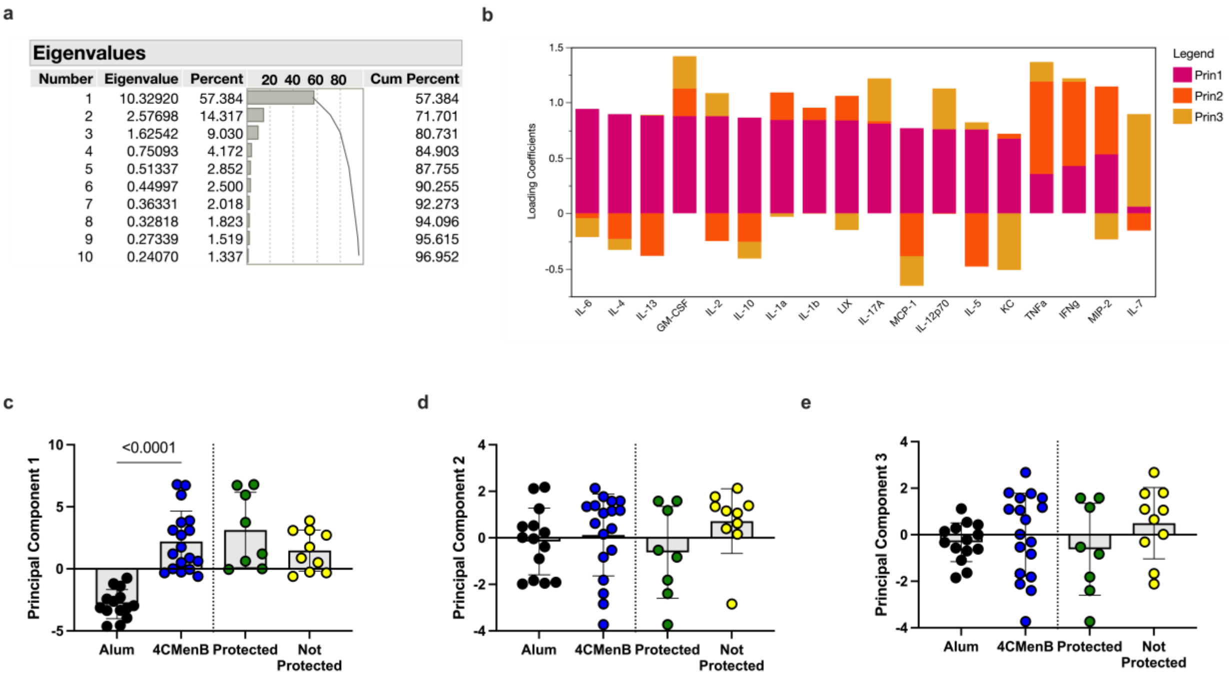

Number	Eigenvalue	Percent	Cum Percent
1	10.32920	57.384	57.384
2	2.57698	14.317	71.701
3	1.62542	9.030	80.731
4	0.75093	4.172	84.903
5	0.51337	2.852	87.755
6	0.44997	2.500	90.255
7	0.36331	2.018	92.273
8	0.32818	1.823	94.096
9	0.27339	1.519	95.615
10	0.24070	1.337	96.952

Figure S16. Flow cytometry T cell frequencies Principal Component Analysis results in 4CMenB-stimulated splenocytes. (**a**) The first ten eigenvalues with individual percentages and cumulative percentages. The first three principal components (PC) explain nearly 45% of the variability of the data. (PC1 = 20.22%, PC2 = 13.169%, and PC3 = 11.484%). (**b**) Stack-plot of loading coefficients on the first 3 principal components. The x-axis shows cell subsets as well as cytokine-producing cells, the y-axis shows the magnitude and direction of the loading coefficients on PC1 (pink), PC2 (orange), and PC3 (yellow) for each variable. Note that individual loadings range between -1 (strongest possible negative correlation) and 1 (strongest possible positive correlation). Scores for PC1 (**c**), PC2 (**d**), and PC3 (**e**) comparing alum (black) and 4CMenB (blue) treated animal (left) and protected (green) and not protected (yellow) 4CMenB-vaccinated animals (right). Unpaired t-tests compared the groups with p-values < 0.05 shown.

a

Eigenvalues

Number	Eigenvalue	Percent	20 40 60 80	Cum Percent
1	11.52537	20.220		20.220
2	7.50651	13.169		33.389
3	6.54560	11.484		44.873
4	5.16015	9.053		53.926
5	3.82132	6.704		60.630
6	2.95642	5.187		65.816
7	2.38406	4.183		69.999
8	2.21579	3.887		73.886
9	1.86868	3.278		77.165
10	1.71356	3.006		80.171

b

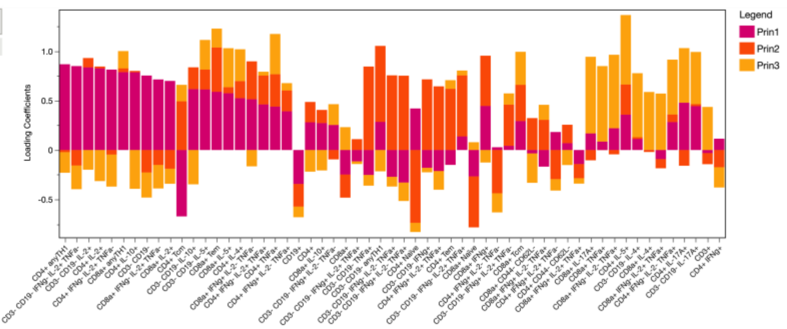

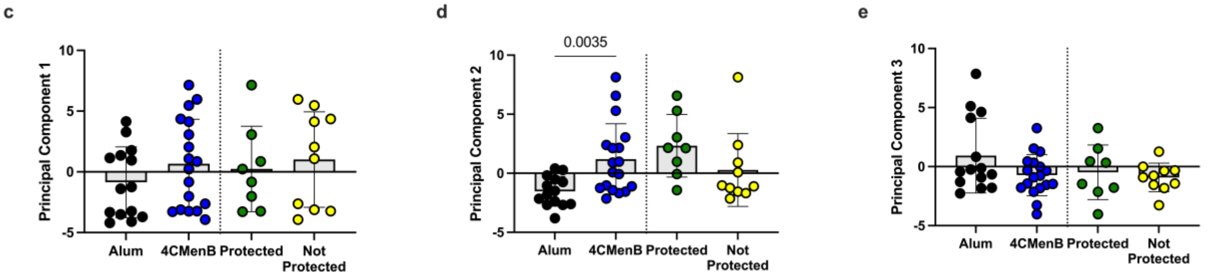

Figure S17. **Flow cytometry T cell total cells per gram of tissue Principal Component Analysis results in 4CMenB-stimulated splenocytes**. (**a**) The first ten eigenvalues with individual percentages and cumulative percentages. The first three principal components (PC) explain nearly 58% of the variability of the data. (PC1 = 34.605%, PC2 = 14.313%, and PC3 = 8.830%). (**b**) Stack-plot of loading coefficients on the first 3 principal components. The x-axis shows cell subsets as well as cytokine-producing cells, the y-axis shows the magnitude and direction of the loading coefficients on PC1 (pink), PC2 (orange), and PC3 (yellow) for each variable. Note that individual loadings range between -1 (strongest possible negative correlation) and 1 (strongest possible positive correlation). Scores for PC1 (**c**), PC2 (**d**), and PC3 (**e**) comparing alum (black) and 4CMenB (blue) treated animal (left) and protected (green) and not protected (yellow) 4CMenB-vaccinated animals (right). Unpaired t-tests compared the groups with p-values < 0.05 shown.

a

Eigenvalues

Number	Eigenvalue	Percent	Cum Percent
1	19.72482	34.605	34.605
2	8.15838	14.313	48.918
3	5.03316	8.830	57.748
4	4.04735	7.101	64.849
5	2.76980	4.859	69.708
6	2.36568	4.150	73.858
7	2.17170	3.810	77.668
8	1.86381	3.270	80.938
9	1.57029	2.755	83.693
10	1.53988	2.702	86.394

b

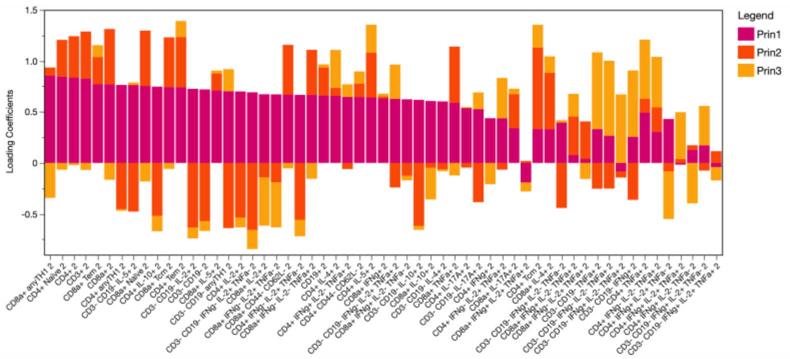

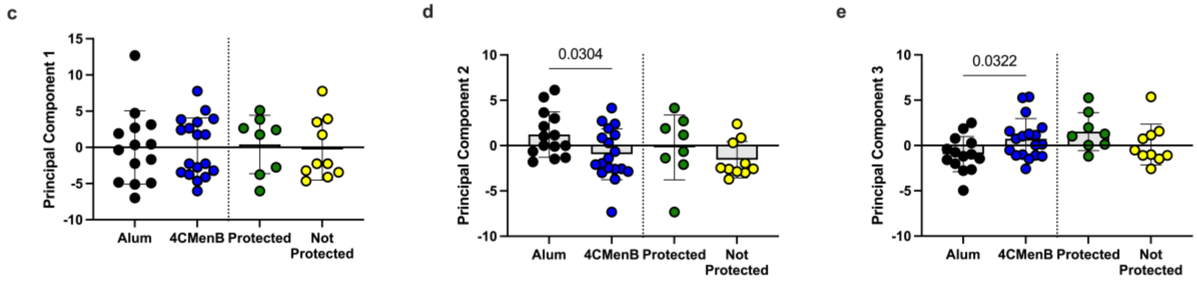

Figure S18. **45-plex Principal Component Analysis results from vaginal lavages**. Left, The first ten eigenvalues with individual percentages and cumulative percentages. Middle, Stack-plot of loading coefficients on the first 3 principal components from each indicated timepoint. The x-axis shows cytokines and chemokines, the y-axis shows the magnitude and direction of the loading coefficients on PC1 (pink), PC2 (orange), and PC3 (yellow) for each variable. Note that individual loadings range between -1 (strongest possible negative correlation) and 1 (strongest possible positive correlation). Right, scores for PC1, PC2, and PC3 comparing alum (black) and 4CMenB (blue) treated animal (left) and protected (green) and not protected (yellow) 4CMenB-vaccinated animals (right). Unpaired t-tests compared the groups with p-values < 0.05 shown. Principal component analyses from each timepoint (prior to infect but post-vaccination, Pre, Top; day 3 post-infection, Middle; day 6 post-infection, Bottom) were performed independent of one another.

Pre

Eigenvalues

Number	Eigenvalue	Percent	20 40 60 80	Cum Percent
1	19.03101	43.252		43.252
2	6.14076	13.956		57.209
3	4.59307	10.439		67.647
4	3.12604	7.105		74.752
5	2.25598	5.127		79.879
6	1.63575	3.718		83.597
7	1.35899	3.089		86.685
8	1.15137	2.617		89.302
9	0.76531	1.739		91.042
10	0.65855	1.497		92.538

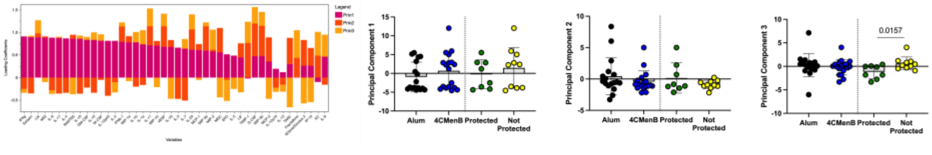

Day 3

Eigenvalues

Number	Eigenvalue	Percent	20 40 60 80	Cum Percent
1	19.68510	46.869		46.869
2	4.94690	11.778		58.648
3	3.00932	7.165		65.813
4	2.87203	6.838		72.651
5	1.87853	4.473		77.124
6	1.67573	3.990		81.113
7	1.56147	3.718		84.831
8	1.03583	2.466		87.297
9	0.97523	2.322		89.619
10	0.91056	2.168		91.787

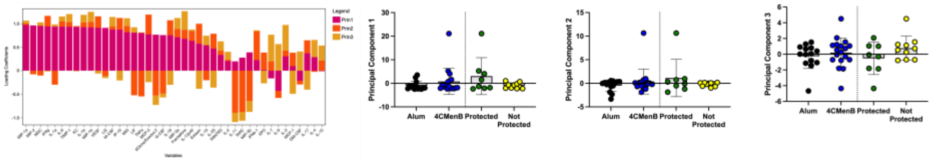

Day 6

Eigenvalues

Number	Eigenvalue	Percent	20 40 60 80	Cum Percent
1	16.68810	38.810		38.810
2	6.54999	15.233		54.042
3	3.85362	8.962		63.004
4	3.63961	8.464		71.468
5	1.99675	4.644		76.112
6	1.66364	3.869		79.981
7	1.50244	3.494		83.475
8	1.35392	3.149		86.623
9	1.06297	2.472		89.095
10	0.81818	1.903		90.998

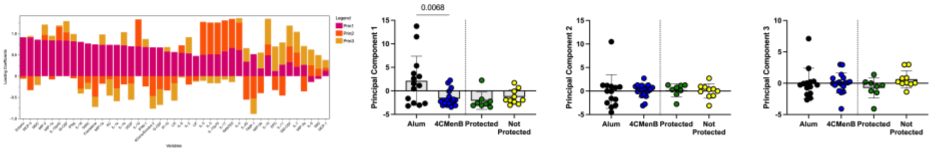

Supplement: Supplemental figures, part 3 — Fig. S13 to S18. [file msphere.00940-24-s0003.pdf]
